# YWHAZ downregulated innate immune responses to RNA viruses by inhibiting the IRF3 signaling pathway

**DOI:** 10.1128/msphere.00038-26

**Published:** 2026-04-03

**Authors:** Shasha Li, Jinyuan Han, Hefei Wang, Jixia Hou, Linhao Wang, Zhengyang Hou, Yaxin Zhang, Xueer Dou, Jingying Xie, Huixia Li, Xiangrong Li, Ruofei Feng

**Affiliations:** 1 Engineering Research Center of Key Technology and Industrialization of Cell-based Vaccine, Ministry of Education, Biomedical Research Center, Northwest Minzu University 66293, Lanzhou, China; 2 Key Laboratory of Biotechnology and Bioengineering of State Ethnic Affairs Commission, Biomedical Research Center, Northwest Minzu University 66293, Lanzhou, China; 3 School of Life Sciences and Engineering, Northwest Minzu University 66293, Lanzhou, China; 4 Gansu University of Traditional Chinese Medicine 381940, Lanzhou, China; Instituto de Biotecnologia/UNAM, Cuernavaca, Morelos, Mexico

**Keywords:** YWHAZ, viral infection, innate immunity, IRF3, type 1 IFN

## Abstract

Tyrosine 3-monooxygenase/tryptophan 5-monooxygenase activation protein zeta polypeptide (YWHAZ) is a member of the YWHA/14-3-3 family. YWHAZ is highly conserved in mammalian cells and generally mediates signal transduction through direct interactions with target proteins. YWHAZ has been reported as a crucial regulator of tumor immunity, inflammation, and apoptosis pathways. However, the precise role of YWHAZ in regulating host antiviral immune responses is not fully understood. Here, our study revealed that YWHAZ negatively regulated type 1 interferon (type 1 IFN) production in response to RNA viruses. Overexpression of YWHAZ inhibited type 1 IFN production triggered by RNA viruses, whereas knockout of YWHAZ increased type 1 IFN production. Mechanistically, YWHAZ interacted with IRF3 and suppressed the nuclear translocation of IRF3 by directly inhibiting IRF3 phosphorylation-dependent dimerization and disrupting the KPNA3-IRF3 interaction. Our findings demonstrated that YWHAZ played a crucial role in regulating IRF3-mediated type 1 IFN production.

**IMPORTANCE:**

The activation of IRF3 induced by RIG-I-like receptors is pivotal for type 1 interferon (IFN) production in antiviral immunity. Virus infection leads to type 1 IFN production through inducing the dimerization and subsequent nuclear translocation of IRF3. Following its activation, IRF3 must be tightly regulated to prevent a dysregulated or excessive immune response. Here, we first found that YWHAZ, a member of the 14-3-3 protein family, is a negative regulator of type 1 IFN production by targeting IRF3 signaling. YWHAZ is bound to IRF3 to inhibit the formation of the TBK1-IRF3 complex, the phosphorylation and dimerization of IRF3, as well as the subsequent nuclear translocation. YWHAZ also impeded the KPNA3-IRF3 interaction by binding to KPNA3, thereby inhibiting IRF3 nuclear translocation. The aa 124-184 in YWHAZ was critical for YWHAZ-mediated suppression of type 1 IFNs. These findings reveal the mechanism by which YWHAZ promotes RNA virus replication, thereby advancing our understanding of how YWHAZ mediates innate immune responses.

## INTRODUCTION

YWHAZ/14-3-3ζ belongs to the tyrosine 3-monooxygenase/tryptophan 5-monooxygenase (YWHA)/14-3-3 activation protein family (1). The members, including YWHAB/14-3-3β, YWHAG/14-3-3γ, YWHAE/14-3-3ε, YWHAZ/14-3-3ζ, YWHAH/14-3-3η, YWHAS/14-3-3σ, and YWHAQ/14-3-3θ/τ, are highly conserved in mammalian systems and generally mediate signal transduction through direct interactions with proteins carrying specific phosphorylated 14-3-3-binding motifs (2). YWHA proteins typically function as homodimers or heterodimers. They act primarily as scaffold proteins, exerting their regulatory functions by protein-protein interactions (3). By binding different ligands, YWHA proteins mediate diverse regulatory processes, such as affecting the phosphorylation status, molecular interactions, modulating subcellular localization, and stability of target proteins (2, 4). YWHA proteins bind phosphorylated serine and threonine residues on target proteins while also being capable of recognizing specific non-phosphorylated hydrophobic peptides (2, 5).

YWHAZ acts as a central hub protein in numerous signal transduction pathways and plays a crucial role in tumor progression (6, 7). YWHAZ activates the PI3K/AKT/Snail signaling to promote the migration and invasion of the glioma cells (8). TMEM65 interacts with YWHAZ to activate PI3K-Akt-mTOR signaling to promote gastric tumorigenesis (6). Emerging evidence suggests that YWHAZ may promote tumor inflammation and modulate immune responses in oral squamous cell carcinoma by activating Stat3 signaling (9). Toll-like receptors (TLRs) and RIG-I-like receptors (RLRs) are two principal classes of pattern recognition receptors (PRRs) that detect RNA viruses and play a vital role in viral pathogenesis and host defense (10). YWHA family members also participate in innate immune regulation, with demonstrated roles in TLR signaling. YWHAZ has opposite effects on TLR2 and TLR4 signaling. It promotes TLR4-mediated NF-κB activation but inhibits TLR2-mediated NF-κB activation (9, 11). Notably, it also positively regulates the TLR3 pathway by facilitating TICAM-1 multimerization (12). However, the role of YWHAZ in the RLR signaling pathway remains poorly studied.

RLR signaling mediated by RIG-I and MDA5 is critical for the elimination of RNA viruses in the antiviral innate immune response. Upon viral infection, PRRs recruit the adaptor proteins, including mitochondrial antiviral signaling protein (MAVS), TIR domain–containing adaptor molecule 1 (TRIF), and stimulator of interferon genes protein (STING), that further interact with Tank-binding kinase 1 (TBK1)/I-κB kinase (IKKε) to form the signal complexes (13). Both TBK1 and IKKε can phosphorylate IRF3 (14). IRF3 is subsequently activated through phosphorylation and dimerization, events that trigger its nuclear translocation. In the nucleus, activated IRF3 binds to the promoters of type 1 interferons (IFN-I) to initiate their transcription (13, 15). IRF3 is widely recognized as a principal and early regulator of IFN-I production during antiviral innate immunity in many cell types. Its transcriptional activity is strictly dependent on dimerization or oligomerization (16). It is reported that multiple proteins are involved in regulating the subcellular signaling for activation of IRF3. Generally, proteins containing a nuclear localization signal (NLS) are transported from the cytoplasm to the nucleus by importin α (also known as karyopherin α, KPNA). KPNA recognizes the NLS on its cargo protein and recruits karyopherin β (KPNB) to facilitate nuclear import (17). Previous studies suggested that KPNA1, KPNA2, KPNA3, and KPNA4 interacted with IRF3 (18, 19). However, knockdown experiments identified KPNA2 as the primary carrier responsible for IRF3 nuclear import (20). Notably, viral proteins such as monkeypox virus protein P2 and African swine fever virus protein DP96R can inhibit the KPNA-IRF3 interaction, thereby disrupting the nuclear localization of IRF3 (19, 21).

Although YWHA proteins are evolutionarily conserved, the specific function of YWHAZ within the RLR signaling pathway remains unexplored. Our previous mass spectrometry analysis revealed that encephalomyocarditis virus (EMCV) infection significantly upregulated YWHAZ expression. Therefore, we aimed to elucidate whether YWHAZ is involved in RLR signaling during RNA virus infection. Here, we demonstrated that YWHAZ downregulated type I interferon production induced by either EMCV or vesicular stomatitis virus (VSV), consequently enhancing viral replication. We further found that YWHAZ inhibited RLR-mediated IRF3 signaling. Mechanistically, YWHAZ suppressed host type 1 IFNs by interacting directly with IRF3 and impeding the nuclear translocation of IRF3 by directly inhibiting IRF3 phosphorylation-dependent dimerization and disrupting the KPNA3-IRF3 interaction. This study identified a novel regulatory role for YWHA proteins within the RLR signaling pathway, thereby highlighting their multifaceted and complex roles in antiviral innate immunity.

## MATERIALS AND METHODS

### Cells and viruses

Human embryonic kidney 293T (HEK-293T) cells were cultured in complete DMEM (Cellmax) supplemented 10% FBS (Gibco) and 1% penicillin/streptomycin (Hyclone). A549 cells and baby hamster Syrian kidney-21 (BHK-21) cells were grown in complete DMEM (Cellmax) containing 10% Newborn Bovine Serum (NBS). All cell lines were maintained at 37°C in a 5% CO_2_ incubator. The PV21 strain of EMCV and the VSV-GFP strain were stored in our laboratory. EMCV strain was amplified in BHK-21 cells. The VSV-GFP strain was amplified in BHK-21 cells. The virus titer was calculated using the Karber method and expressed as 50% tissue culture infectious dose per milliliter (TCID_50_).

### Reagents and antibodies

Polyinosinic-polycytidylic acid [poly(I:C)] was purchased from InvivoGen (USA). Anti-Flag rabbit polyclonal antibody, anti-Flag mouse monoclonal antibody, anti-β-actin rabbit polyclonal antibody, anti-HA rabbit polyclonal antibody, anti-YWHAZ rabbit polyclonal antibody, anti-YWHAZ mouse monoclonal antibody, anti-MDA5 rabbit polyclonal antibody, anti-MAVS rabbit polyclonal antibody, anti-TBK1 rabbit polyclonal antibody, anti-IRF3 rabbit polyclonal antibody, anti-KPNA2 mouse monoclonal antibody, anti-KPNA3 mouse monoclonal antibody, and anti-KPNA4 rabbit polyclonal antibody were purchased from Proteintech. Anti-phospho-TBK1 rabbit monoclonal antibody, anti-phospho-IRF3 rabbit monoclonal antibody, anti-IRF3 mouse monoclonal antibody, and anti-Myc mouse monoclonal antibody were purchased from Cell Signaling Technology.

### Plasmids

The firefly luciferase reporter plasmid IFN-β-Luc, containing the murine IFN-β promoter region (−125 to +55), was kindly provided by the Lanzhou Veterinary Research Institute, Chinese Academy of Agricultural Sciences. The reporter plasmid pRL-TK (RL stands for Renilla luciferase, and TK stands for thymidine kinase) was purchased from Promega Corporation (Madison, WI). The complete CDS sequence of the YWHAZ gene (Gene ID: 7534) was cloned into three eukaryotic expression vectors: *pCAGGS-Myc*, *pCMV-3tag-3a*, and *pcDNA3.1-Myc-His*. This cloning generated the plasmids *pCAGGS-YWHAZ-Myc*, *pCMV-YWHAZ-Flag*, and *pcDNA3.1-YWHAZ-His*. In the overexpression experiments, the corresponding empty vector plasmids (marked as EV in Figures) served as negative controls for *pCAGGS-YWHAZ-Myc*, *pCMV-YWHAZ-Flag*, and *pcDNA3.1-YWHAZ-Myc*. The point mutations of YWHAZ, including YWHAZ(S58A)-Flag, YWHAZ(MMW)-Flag, YWHAZ(WMW)-Flag, YWHAZ(S58E)-Flag, and Flag-IRF3(5A), were generated by the PCR mutagenesis method (22) using the YWHAZ-Flag plasmid as a template. Truncation mutations of YWHAZ, including YWHAZ(Δ1-62)-His, YWHAZ(Δ63-123)-His, YWHAZ(Δ124-184)-His, and YWHAZ(Δ185-246)-His, were generated by the PCR mutagenesis method (22) using the YWHAZ-His plasmid as a template. HA-TBK1, HA-IRF3, Flag-IRF3, and Flag-IRF3(5D) expressing plasmids were purchased from Public Protein/Plasmid Library. These plasmids were transfected into different cells using Lipofectamine 2000 (Invitrogen) at a ratio of 1:1-2:1 (Lipofectamine 2000/DNA), according to Lipofectamine 2000 Reagent instruction.

### Quantitative real-time PCR

Total RNAs were extracted from cells using *TransZol* Up Plus RNA Kit (TransGen Biotech), and cDNA was synthesized using the Hiscript II RT SuperMix for qPCR (+gDNA wiper) kit (Vazyme). Quantitative real-time PCR (Q-PCR) was performed using ChamQ Universal SYBR qPCR Master Mix (Vazyme) on the ABI 7500 Real-Time PCR system. The glyceraldehyde-3-phosphate dehydrogenase (GAPDH) gene was used as an internal control. The relative expression levels were calculated using the 2^−ΔΔCT^ method (23). Q-PCR primers were synthesized by GenScript Biotech Corporation (Nanjing, China), and primer sequences are listed in Table 1.

**TABLE 1:** qPCR primers used in this study

Primer	Sequence (5′−3′)
GAPDH-F	CGGGAAGCTTGTGATCAATGG
GAPDH-R	GGCAGTGATGGCATGGACTG
IFN-β-F	GACATCCCTGAGGAGATTAAG
IFN-β-R	ATGTTCTGGAGCATCTCATAG
ISG15-F	GGACAAATGCGACGAACC
ISG15-R	CCCGCTCACTTGCTGCTT
ISG54-F	ACGGTATGCTTGGAACGATTG
ISG54-R	AACCCAGAGTGTGGCTGATG
ISG56-F	CTTGAGCATCCTCGGGTTCATC
ISG56-R	AAGTCAGCAGCCAGGTTTAGGG
YWHAZ-F	CCTGCATGAAGTCTGTAACTGAG
YWHAZ-R	GACCTACGGGCTCCTACAACA

### Cell viability assay

Cells’ viability was assessed using Cell Counting Kit-8 (CCK-8, Meilunbio) following the manufacturer’s protocol. Briefly, WT and YWHAZ^−/−^ HEK-293T cells were seeded in 96-well culture plates and cultured for 48 h. Then, the medium was replaced with 100 µL fresh medium containing 10 µL of CCK-8 solution. After incubation at 37°C for 0.5–4 hours (h), the absorbance at 450 nm was measured using a microplate reader (Thermo).

### RNA interference (RNAi) assay

The small interfering RNA (siRNA) used in this study was as follows: IRF3-siRNA, AGAGGCTCGTGATGGTCAA. siRNAs were designed and synthesized by Suzhou Ribo Life Science Co., Ltd. The siRNAs were transfected using Lipofectamine 3000 (Invitrogen) according to Lipofectamine 3000 Reagent instructions. At 12 h after siRNA (NC) or siRNA targeting IRF3 (IRF3-siRNA) transfection, HEK-293T cells were transfected with EV (*pCMV-3Tag-3a*) or *pCMV-YWHAZ-Flag* plasmid*s* for another 12 h. Then, cells were infected with EMCV or VSV-GFP. The siRNA efficiency and expression of the immune cytokines in cells were evaluated by qPCR. The supernatants were collected and used in the 50% tissue culture-infective dose assay (TCID_50_) to calculate virus yields (24).

### Dual-luciferase reporter assays

HEK-293T cells cultured in 48-well plates were cotransfected with 50 ng of reporter plasmid (IFN-β), 5 ng of pRL-TK, together with the indicated empty vector or YWHAZ-Myc-expressing protein. At 24 h post-transfection (hpt), cells were transfected with poly(I:C) for 12 h. Cells were lysed with lysis buffer for 15 min at room temperature, and a dual-luciferase reporter assay kit (Promega) was employed to measure firefly and *Renilla* luciferase activities following the manufacturer’s protocol. Relative luciferase activity was analyzed by normalization of firefly luciferase to *Renilla* luciferase activity.

### Immunoblot analysis and coimmunoprecipitation

Cells after transfection were lysed with RIPA buffer (high) (Solarbio) supplemented with protease inhibitor PMSF. Then, the coimmunoprecipitation (Co-IP) and immunoblot analysis were performed as previously described (25). Immunoprecipitates or whole-cell lysates (WCLs) were subjected to SDS-PAGE, transferred onto polyvinylidene fluoride (PVDF) membranes (Millipore), and then blotted with specific primary antibodies. After five washes, the membranes were incubated with appropriate HRP-conjugated secondary antibodies. Proteins were visualized with Clarity Western ECL substrate (Bio-Rad). The expression of β-actin was used as a loading control.

### Establishment of a YWHAZ knockout HEK-293T cell line using the CRISPR/Cas9 system

CRISPR-Cas9-mediated ablation of the YWHAZ gene was achieved using CRISPR-Cas9 RNP (provided by Haixing Bioscience) containing expression cassettes for hSpCas9 and chimeric guide RNA. To target exon 5 of the YWHAZ gene, two guide RNA sequences of TAAGGAGTCCATGCCAGAGAAGG and AACATCCCTGTACACAATATTGG were selected through the http://crispor.tefor.net website. Plasmid containing the guide RNA sequence was electrotransfected into cells using the Neon transfection system according to the manufacturer’s instructions (ThermoFisher Scientific). After 2 days, single colonies were transferred into 96-well plates. To identify insertions or deletions (indels) in YWHAZ-targeted clones, genomic DNA was extracted using a Quick-DNA Miniprep kit (Zymo Research). PCR amplification was performed with 2× Taq Master Mix (Dye Plus; Vazyme, P112) using primers flanking the exon: Forward: 5′-CCCATCCCCAACACAATGATCTAA-3′; Reverse: 5′-GGGAGCTTTCTCCTGGTACAC-3′. Plasmids from 8 to 10 single colonies were isolated and sequenced via Sanger sequencing (GENEWIZ, China). Clones with mutations in both alleles were chosen for further studies.

### Immunofluorescence

HEK293T cells were cultured in Nunc glass-bottom dishes and transfected with the indicated plasmids for 24 h. Cells were transfected with poly(I:C) for 12 h and fixed with 4% paraformaldehyde at room temperature for 15 min. Then, cells were permeabilized with PBS containing 0.2% Triton X-100 and blocked with PBS containing 5% BSA for 1 h at room temperature. The cells were immunostained with the indicated primary antibodies at 4°C overnight, followed by incubation with the corresponding secondary antibodies (Invitrogen) at 37°C for 1 h. The nuclei were stained with DAPI (Abcam). The cells were imaged using a Zeiss LSM900 confocal microscope and analyzed by ZEN imaging software.

### Cytosolic and nuclear protein fractionation

Nuclear and cytoplasmic proteins were extracted using the Nuclear/Cytosol Fractionation Kit (BioVision) according to the manufacturer’s instructions. The expression of IRF3, YWHAZ, Histone 3, and HSP90 was analyzed by western blotting. Histone 3 and HSP90 were analyzed as fraction loading controls.

### Statistical analysis

Statistical analyses were performed using GraphPad Prism version 8.00 for Windows (GraphPad Software). An unpaired two-tailed Student’s *t*-test was used for comparisons between two groups. For multiple comparisons involving a single factor, one-way ANOVA was conducted, followed by Tukey’s multiple comparisons test. Data are obtained from three independent experiments and represented as mean ± standard deviation (SD). Statistical significance was defined as *P* < 0.05.

## RESULTS

### YWHAZ inhibited type 1 IFN response to RNA virus infection

**Fig 1:**
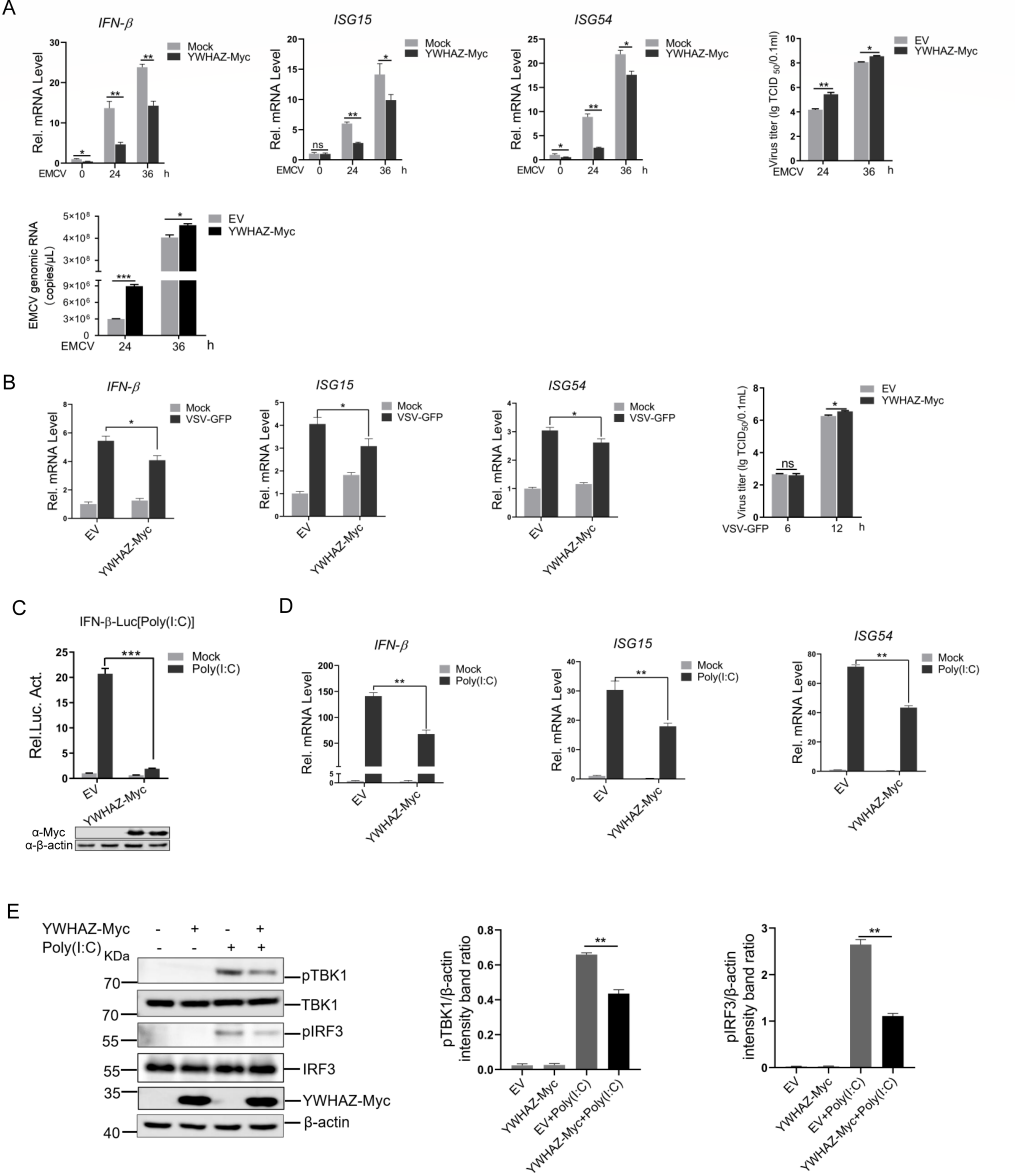
YWHAZ inhibited IFN-β and ISGs expression to promote viral replication. (**A**) HEK-293T cells were transfected with the empty vector (EV) *pCAGGS-Myc* or *pCAGGS-YWHAZ-Myc* plasmid*s*. At 24 hpt, cells were infected with EMCV (MOI = 0.01) or mock-infected for 24 h or 36 h. The cells transfected with an empty vector were used as a negative control. The cells were used to analyze the mRNA level of IFN-β, ISG15, and ISG54 by Q-PCR. The supernatants were used to test the viral RNA copy numbers and titers of EMCV by Q-PCR and TCID_50_ assay. EV, empty vector. Representative data from three independent experiments. Data were shown as mean ± SD (*n* = 3). One-way ANOVA with Tukey’s multiple-comparison test was performed. ns, not significant. *P < 0.05; **P < 0.01; ***P < 0.001. (**B**) HEK-293T cells were transfected with the empty vector *pCAGGS-Myc* or *pCAGGS-YWHAZ-Myc* plasmid*s*. At 24 hpt, cells were infected with VSV-GFP (MOI=0.001) for 6 h or 12 h. The cells transfected with an empty vector were used as a negative control. The cells were used to analyze the mRNA levels of IFN-β, ISG15, and ISG54 by Q-PCR, and the supernatants were used to test the titers of VSV-GFP by the TCID_50_ assay. EV, empty vector. Representative data from three independent experiments. Data were shown as mean ± SD (*n* = 3). Student’s t test was performed. ns, not significant. *P < 0.05. (**C**) HEK-293T cells were cotransfected with the IFN-β-Luc reporter, pRL-TK reporter, and the indicated empty vector *pCAGGS-Myc* or *pCAGGS-YWHAZ-Myc* plasmids. At 24 hpt, cells were transfected with poly(I:C) for 12 h. The cells transfected with an empty vector were used as a negative control. The cells were collected to analyze the luciferase activity (upper panels), and the expression of YWHAZ-Myc and β-actin was analyzed by western blotting (lower panels). EV, empty vector. Representative data from three independent experiments. Data were shown as mean ± SD (*n* = 3). Student’s t test was performed. ***P < 0.001. (**D and E**) HEK-293T cells were transfected with the empty vector *pCAGGS-Myc* or *pCAGGS-YWHAZ-Myc* plasmid*s*. At 24 hpt, cells were transfected with poly(I:C) for 12 h. The cells transfected with an empty vector were used as a negative control. Cells were collected and used for western blotting (**E**) and Q-PCR (**D**) assays, respectively. (**D**) The mRNA levels of IFN-β, ISG15, and ISG54 were analyzed by Q-PCR. EV, empty vector. (**E**) The TBK1, phosphorylated TBK1, IRF3, phosphorylated IRF3, YWHAZ-Myc, and β-actin were analyzed by western blotting. Band intensity of phosphorylated TBK1 and IRF3 protein was determined by densitometric analysis using ImageJ after normalization to β-actin expression. Representative data from three independent experiments. Data were shown as mean ± SD (*n* = 3). Student’s *t* test was performed. **P < 0.01.

YWHAZ is a molecular mass of approximately 30 kDa scaffolding protein that serves as a key hub in multiple signal transduction pathways (7). In our previous study, mass spectrometry analysis showed that EMCV infection upregulated YWHAZ expression. HEK-293T cells, widely used due to their high transfection efficiency, are also permissive to infection by RNA viruses such as EMCV and VSV. To investigate the role of YWHAZ in immune response induced by RNA viruses, we examined the effect of YWHAZ overexpression on EMCV-induced type 1 IFN expression in HEK-293T cells. Q-PCR analysis indicated that YWHAZ expression suppressed the mRNA level of IFN-β, ISG15, and ISG54 induced by EMCV (Fig. 1A). Furthermore, overexpression of YWHAZ in HEK-293T cells significantly increased EMCV viral RNA copy numbers and viral titers at both 24 and 36 h post-infection (hpi) (Fig. 1A). Next, we used a VSV infection model to evaluate the effect of YWHAZ on type 1 IFN production. As shown in Fig. 1B, overexpression of YWHAZ reduced VSV-GFP-induced IFN-β, ISG15, and ISG54 mRNA expression. Concurrent measurement of viral titers in the supernatant revealed that YWHAZ overexpression increased VSV-GFP proliferation at 12 hpi (Fig. 1B). Our findings indicated that YWHAZ increased the proliferation of EMCV and VSV by inhibiting the expression of IFN-β and ISGs. Notably, the inhibitory effect on EMCV proliferation was more pronounced than on VSV. Various studies have demonstrated that MDA5 is mostly responsible for sensing infection of picornaviruses, such as encephalomyocarditis virus (EMCV), while infection of VSV is mainly sensed by RIG-I (26–28). Our data suggested that YWHAZ may regulate both MDA5- and RIG-I-mediated type 1 interferon production, and that its effect on the MDA5 signaling pathway is more significant. To confirm the function of YWHAZ in regulating RLR signaling, we used the RIG-I/MDA5 agonist poly(I:C) to identify the effect of YWHAZ on RLR-mediated type 1 interferon response. The results indicated that IFN-β promoter activity was efficiently induced in cells stimulated with poly(I:C), but was significantly lower in cells expressing YWHAZ-Myc (Fig. 1C). Similarly, YWHAZ significantly decreased the expression of IFN-β and ISG15, ISG54 mRNA triggered by poly(I:C) in HEK-293T cells (Fig. 1D). To assess the changes in RLR-mediated signaling cascades upon poly(I:C) stimulus in cells expressing YWHAZ-Myc, we analyzed the phosphorylation levels of TBK1 and IRF3, which are the hallmarks of IFNs pathway activation. YWHAZ expression decreased the phosphorylation of TBK1 and IRF3 induced by poly(I:C) in HEK-293T cells (Fig. 1E). Altogether, these results indicated that YWHAZ negatively regulated type 1 IFN response upon RNA virus infection.

### Deletion of YWHAZ promoted type 1 IFN response

**Fig 2:**
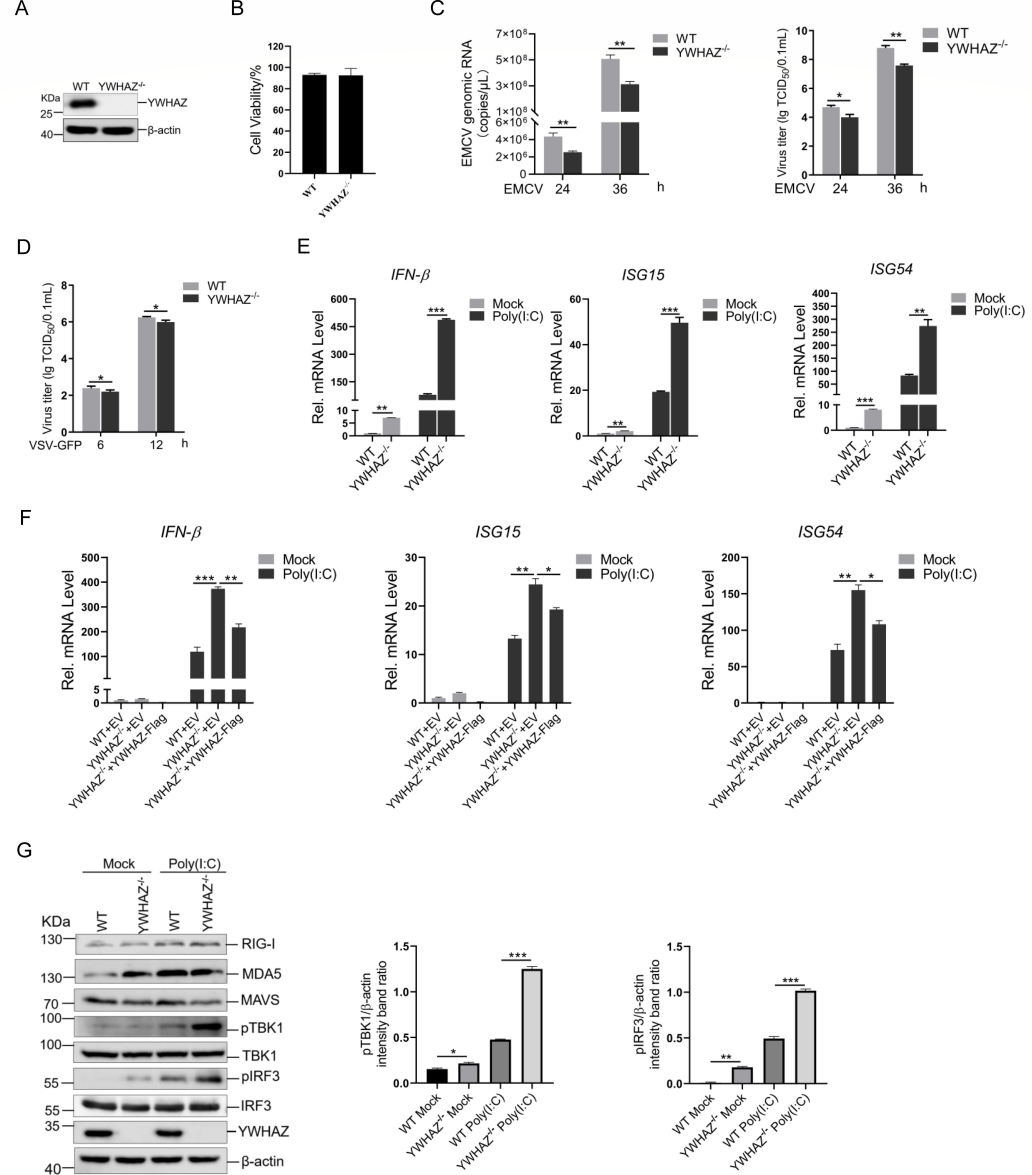
YWHAZ deficiency increased type 1 IFN and ISGs expression and suppressed RNA virus replication. (**A**) WT and YWHAZ^−/−^ HEK-293T cells were separately harvested and used for western blotting to detect the protein levels of YWHAZ and β-actin. (**B**) WT and YWHAZ^−/−^ HEK-293T cells were separately seeded in 96-well plates and cultured for 48 h. Cell viability was assessed using Cell Counting Kit-8 (CCK-8, Meilunbio) following the manufacturer’s protocol. (**C**) WT and YWHAZ^−/−^ HEK-293T cells were infected with EMCV (MOI = 0.01) or mock-infected for 24 h or 36 h. Viral titer and genomic copy numbers of EMCV were determined by TCID_50_ assay and Q-PCR, respectively. Representative data from three independent experiments. Data were shown as mean ± SD (*n* = 3). Student’s *t* test was performed. *P < 0.05; **P < 0.01. (**D**) WT and YWHAZ^−/−^ HEK-293T cells were infected with VSV-GFP (MOI = 0.001) or mock-infected for 6 h or 12 h. Viral titer of VSV-GFP was determined by TCID_50_ assay. Representative data from three independent experiments. Data were shown as mean ± SD (*n* = 3). Student’s *t* test was performed. *P < 0.05. (**E**) WT and YWHAZ^−/−^ HEK-293T cells were transfected with poly(I:C) for 12 h, and then the mRNA levels of IFN-β, ISG15, and ISG54 were analyzed by Q-PCR. Representative data from three independent experiments. Data were shown as mean ± SD (*n* = 3). Student’s *t* test was performed. **P < 0.01; ***P < 0.001. (**F**) WT and YWHAZ^−/−^ HEK-293T cells were transfected with EV (*pCMV-3tag-3a*) or YWHAZ-Flag expressing plasmids for 12 h, followed by stimulation with poly(I:C) for an additional 12 h. The mRNA levels of IFN-β, ISG15, and ISG54 were analyzed by Q-PCR. EV, empty vector. Representative data from three independent experiments. Data were shown as mean ± SD (*n* = 3). One-way ANOVA with Tukey’s multiple-comparison test was performed. *P < 0.05; **P < 0.01; ***P < 0.001. (**G**) WT and YWHAZ^−/−^ HEK-293T cells were transfected with poly(I:C) for 12 h, and then the TBK1, phosphorylated TBK1, IRF3, phosphorylated IRF3, YWHAZ protein, and β-actin were analyzed by western blotting. Band intensity of phosphorylated TBK1 and IRF3 proteins was determined by densitometric analysis using ImageJ after normalization to β-actin expression. Data were shown as mean ± SD (*n* = 3). Student’s *t* test was performed. *P < 0.05; **P < 0.01; ***P < 0.001.

To confirm the function of YWHAZ, a YWHAZ gene knockout (KO) HEK-293T cell line (YWHAZ^−/−^) was generated using CRISPR-Cas9 to investigate the function of YWHAZ. Western blotting analysis confirmed the deletion of YWHAZ in YWHAZ^−/−^ HEK-293T cells (Fig. 2A). Knockout of YWHAZ did not significantly affect HEK-293T cell viability and growth (Fig. 2B). The wild type (WT) and YWHAZ^−/−^ HEK-293T cells were infected with EMCV for 24 h or 36 h, and viral titers and RNA copy numbers of EMCV were significantly lower in YWHAZ^−/−^ cells than in WT cells (Fig. 2C). Similarly, YWHAZ^−/−^ HEK-293T cells infected with VSV-GFP exhibited significantly lower VSV viral titers than WT cells (Fig. 2D). Additionally, we measured mRNA expression of IFN-β, ISG15, and ISG54 induced by poly(I:C) in YWHAZ^−/−^ HEK-293T cells (Fig. 2E). Consistent with these observations, mRNA levels of IFN-β, ISG15, and ISG54 were significantly elevated in YWHAZ^−/−^ cells following poly(I:C) treatment (Fig. 2E). We then restored YWHAZ by transiently transfecting YWHAZ-Flag into the knockout HEK-293T cells. Q-PCR analysis showed that ectopic YWHAZ expression reversed the enhanced induction of IFN-β, ISG15, and ISG54 mRNAs caused by YWHAZ deletion (Fig. 2F). Likewise, phosphorylation levels of TBK1 and IRF3 were increased in YWHAZ^−/−^ HEK-293T cells (Fig. 2G). The results showed that YWHAZ knockout enhances type 1 IFN production.

### YWHAZ interacted with IRF3

**Fig 3:**
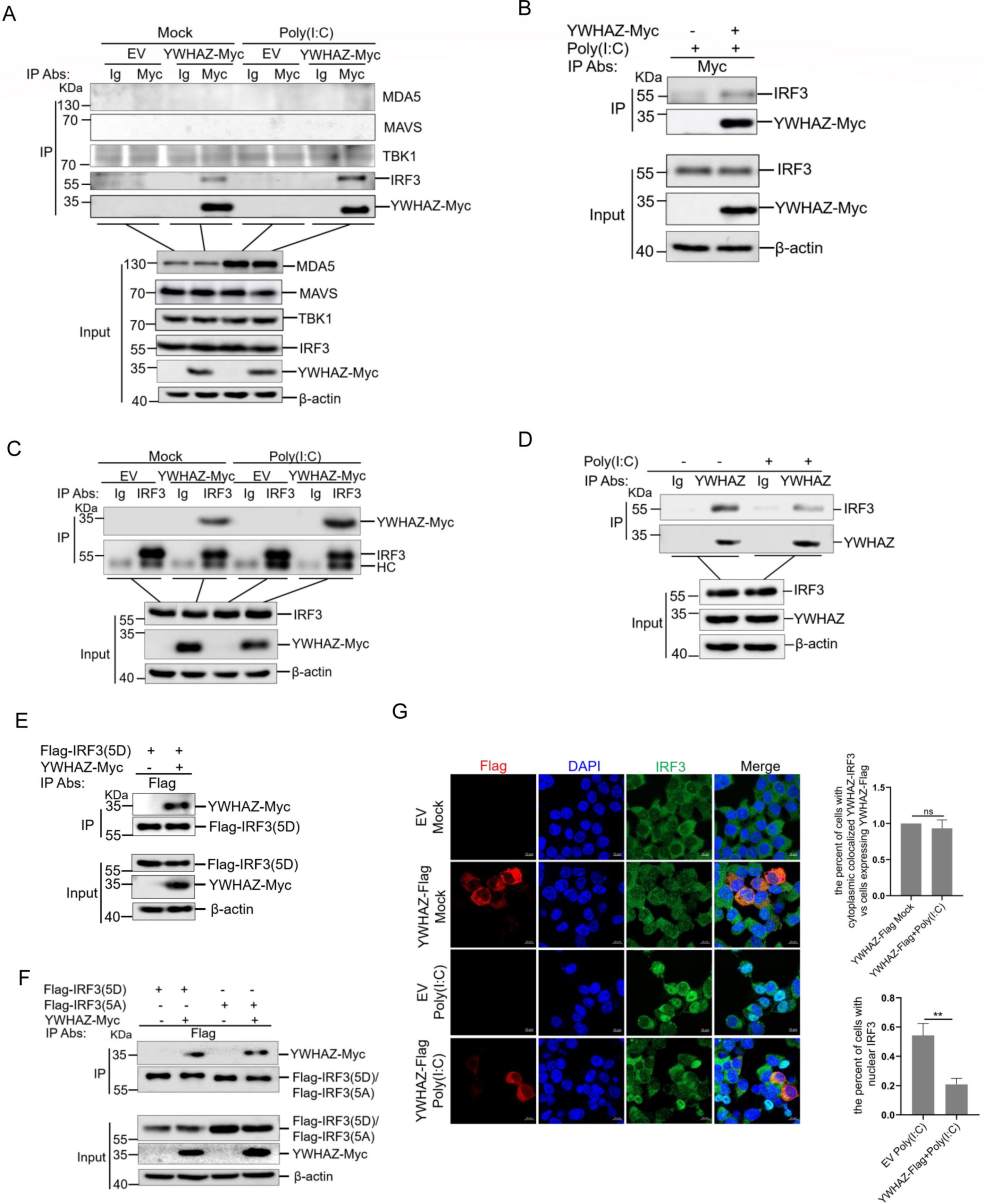
YWHAZ interacted with IRF3. (**A**) HEK-293T cells were transfected with the EV (*pCAGGS-Myc*) or *pCAGGS-YWHAZ-Myc* plasmid*s*. At 24 hpt, cells were transfected with poly(I:C) for 12 h and then were harvested and used for Co-IP with control immunoglobulin G (IgG) or anti-Myc antibodies. The cells transfected with an empty vector were used as a negative control. WCLs and immunoprecipitates were analyzed by western blotting with the indicated antibodies. EV, empty vector. Representative data from three independent experiments. (**B**) A549 cells were transfected with the EV (*pCAGGS-Myc*) or YWHAZ-Myc expressing plasmid*s*. At 24 hpt, cells were transfected with poly(I:C) for 12 h. Cells were harvested and used for the Co-IP with anti-Myc antibody. WCL and immunoprecipitates were analyzed by western blotting with the indicated antibodies. Representative data from three independent experiments. (**C**) HEK-293T cells were transfected with the EV (*pCAGGS-Myc*) or *pCAGGS-YWHAZ-Myc* plasmid*s*. At 24 hpt, cells were transfected with poly(I:C) for 12 h. The cells transfected with an empty vector were used as a negative control. Cells were harvested and used for the Co-IP with control IgG or anti-IRF3 antibodies. WCL and immunoprecipitates were analyzed by western blotting with the indicated antibodies. EV, empty vector. Representative data from three independent experiments. (**D**) HEK-293T cells were transfected with poly(I:C) for 12 h. Cells were harvested and used for the Co-IP with control IgG or anti-YWHAZ antibodies. WCL and immunoprecipitates were analyzed by western blotting with indicated antibodies. Representative data from three independent experiments. (**E and F**) HEK-293T cells were cotransfected with the EV (*pCAGGS-Myc*) or pCAGGS-YWHAZ-Myc plasmids, together with the plasmids expressing Flag-IRF3(5D) (**E**) or Flag-IRF3(5D) or Flag-IRF3(5A) (**F**). The cells transfected with an empty vector and Flag-IRF3(5D) or Flag-IRF3(5A) were used as a negative control. At 36 hpt, cells were harvested and used for the Co-IP with anti-Flag antibodies. WCL and immunoprecipitates were analyzed by western blotting with the indicated antibodies. Representative data from three independent experiments. (**G**) HEK-293T cells were transfected with EV (*pCMV-3Tag-3a*) or YWHAZ-Flag expressing plasmids for 24 h followed by transfection of poly(I:C) for 12 h. Cells were then immunostained with anti-Flag and anti-IRF3 antibodies. IRF3 is in green, YWHAZ-Flag is in red, and cell nuclei were stained with DAPI (blue). The percentage of YWHAZ-Flag-expressing cells that showed cytoplasmic colocalization of YWHAZ and IRF3 was quantified. Moreover, the translocation percentage of IRF3 in poly(I:C)-treated groups was calculated and compared. Scale bars, 10 μm. Data were shown as mean ± SD (*n* = 3). Student’s *t* test was performed. ns, not significant. **P < 0.01.

Our data suggested that YWHAZ protein plays a vital role in the RLR signaling pathway. To explore how YWHAZ protein inhibited IFN-β production during viral infection, we investigated the interactions between YWHAZ-Myc and RLR signaling molecules, including MDA5, MAVS, TBK1, and IRF3 in HEK-293T cells. The IgG (antibody) Ab was used as a negative control. Co-immunoprecipitation assays showed that anti-Myc Ab-immunoprecipitated protein complexes contained IRF3, but not MDA5, MAVS, and TBK1 (Fig. 3A). YWHAZ interacted with IRF3, regardless of whether cells were stimulated with poly(I:C) or not (Fig. 3A). A549 cells are also capable of generating functional responses to viral infections by producing type 1 interferons and inflammatory factors (29). To determine whether the observed interaction was specific to HEK-293T cells, we examined the association between YWHAZ and IRF3 in A549 cells. As is shown in Fig. 3B, A549 cells were transfected with YWHAZ-Myc or empty vector, followed by transfection with poly(I:C). Co-IP experiments indicated that YWHAZ-Myc interacted with endogenous IRF3 in A549 cells (Fig. 3B). Additionally, we performed a reverse Co-IP using an anti-IRF3 antibody that exhibits high immunoprecipitation efficiency in HEK-293T cells. The results showed that YWHAZ-Myc protein was efficiently coimmunoprecipitated with IRF3 (Fig. 3C). To determine whether there is an interaction between endogenous YWHAZ and IRF3, we performed immunoprecipitation using an antibody against YWHAZ in poly(I:C)-unstimulated or stimulated cells, with Ig Ab serving as a negative control (Fig. 3D). The results showed that endogenous YWHAZ and IRF3 interacted, and this interaction was stronger in unstimulated cells (Fig. 3D). IRF3(5D) is constitutively active and widely used to induce type 1 IFNs (30). IRF3(5A) is a phosphorylation-deficient mutant that is largely retained in the cytoplasm (31). To assess the interaction between YWHAZ and IRF3 in different phosphorylation states, we performed Co-IP experiments using Flag-IRF3(5D) and Myc-tagged YWHAZ. We found that YWHAZ interacted with Flag-IRF3(5D) in HEK-293T cells (Fig. 3E). The interaction between IRF3(5A) with YWHAZ-Myc was also detected in cells co-transfected with Flag-IRF3(5A) and Myc-tagged YWHAZ (Fig. 3F). These data indicated that IRF3 phosphorylation does not alter its association with YWHAZ. To investigate the cellular localization of YWHAZ and IRF3, YWHAZ-Flag was transfected into HEK-293T cells, followed by transfection with poly(I:C). Confocal microscopy analysis demonstrated that YWHAZ-Flag colocalized with endogenous IRF3 in HEK-293T cells (Fig. 3G). Together, these results demonstrated that YWHAZ interacted with IRF3.

### YWHAZ disrupted IRF3-mediated signaling

**Fig 4:**
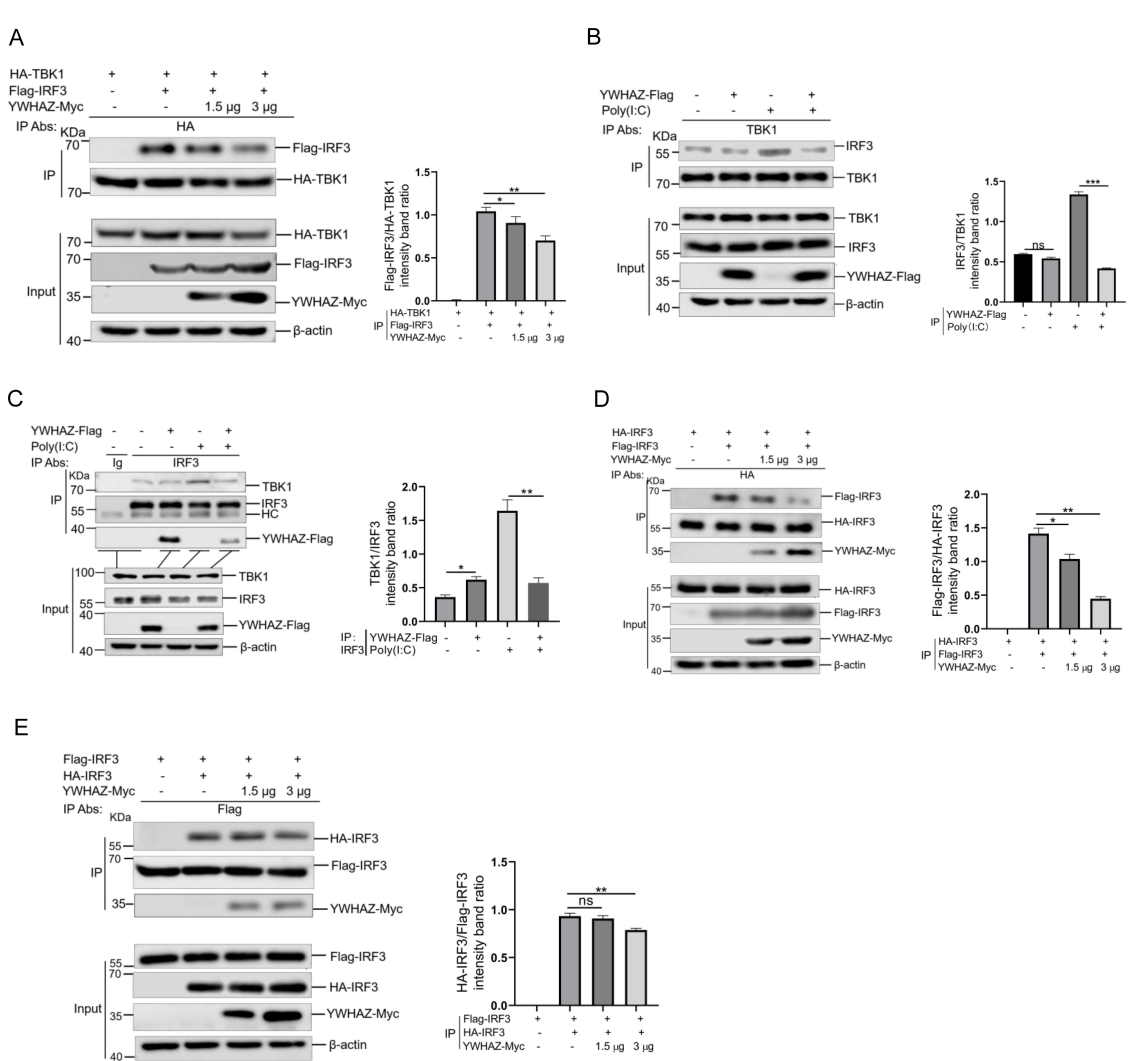
YWHAZ suppressed both TBK1-IRF3 complex assembly and IRF3 dimerization. (**A**) HEK-293T cells were cotransfected with HA-TBK1, Flag-IRF3, and YWHAZ-expressing plasmids or EV (*pCAGGS-Myc*). At 36 hpt, cells were harvested and used for the Co-IP with anti-HA antibody. WCL and immunoprecipitates were analyzed by western blotting with the indicated antibodies. Band intensity of Flag-IRF3 protein was determined by densitometric scanning using ImageJ after normalization to HA-TBK1 expression in the immunoprecipitation complexes. Data were shown as mean ± SD (*n* = 3). One-way ANOVA with Tukey’s multiple-comparison test was performed. *P < 0.05; **P < 0.01. (**B**) HEK-293T cells were transfected with the EV *pCMV-3Tag-3a* or *pCMV-YWHAZ-Flag* plasmid*s*. At 24 hpt, cells were transfected with poly(I:C) for 12 h. The cells transfected with an empty vector were used as a negative control. Cells were harvested and used for the Co-IP with anti-TBK1 antibody. WCL and immunoprecipitates were analyzed by western blotting with the indicated antibodies. Band intensity of IRF3 protein was determined by densitometric scanning using ImageJ after normalization to TBK1 expression in the immunoprecipitation complexes. Data were shown as mean ± SD (*n* = 3). Student’s *t* test was performed. ns, not significant. ***P < 0.001. (**C**) A549 cells were transfected with the empty vector *pCMV-3Tag-3a* or *pCMV-YWHAZ-Flag* plasmid*s*. At 24 hpt, cells were transfected with poly(I:C) for 12 h. The cells transfected with an empty vector were used as a negative control. Cells were harvested and used for the Co-IP with control IgG or anti-IRF3 antibodies. WCL and immunoprecipitates were analyzed by western blotting with the indicated antibodies. Band intensity of TBK1 protein was determined by densitometric scanning using ImageJ after normalization to IRF3 expression in the immunoprecipitation complexes. Representative data from three independent experiments. Data were shown as mean ± SD (*n* = 3). Student’s *t* test was performed. *P < 0.05; **P < 0.01. (**D**) HEK-293T cells were cotransfected with HA-IRF3, Flag-IRF3, and YWHAZ-expressing plasmids or EV (*pCAGGS-Myc*). At 36 hpt, cells were harvested and used for the Co-IP with anti-HA antibody. WCL and immunoprecipitates were analyzed by western blotting with the indicated antibodies. Band intensity of Flag-IRF3 protein was quantified using ImageJ software and normalized to that of HA-IRF3 in the immunoprecipitation complexes. Data were shown as mean ± SD (*n* = 3). One-way ANOVA with Tukey’s multiple-comparison test was performed. * *P* < 0.05; ** *P* < 0.01. (**E**) The same experiment performed as in (**D**) using an anti-Flag antibody for IP.

The transcription factor IRF3 is responsible for initiating the type 1 IFNs production and critical for innate immune responses. Following viral infection, the kinases TBK1 and IKKε phosphorylate IRF3, leading to its dimerization and subsequent translocation into the nucleus (14). These IRF3 dimers then initiate the transcription of target genes such as IFN-β. As YWHAZ interacted with IRF3, we sought to investigate whether YWHAZ could interfere with the complex of IRF3 with TBK1. Flag-IRF3 and HA-TBK1, together with Myc-tagged YWHAZ, were cotransfected into HEK-293T cells, and a Co-IP assay was then conducted using the anti-HA antibody. Results suggested that YWHAZ overexpression impaired the association between TBK1 and IRF3 (Fig. 4A). Furthermore, endogenous TBK1-IRF3 complexes were examined. Co-IP analysis indicated that the form of endogenous TBK1-IRF3 complexes was disrupted significantly in cells expressing YWHAZ-Flag (Fig. 4B). Consistently, YWHAZ overexpression also suppressed the interaction of TBK1 and IRF3 in A549 cells (Fig. 4C). These data suggested that YWHAZ disrupted the formation of TBK1-IRF3 complexes, which aligns with its role in negatively regulating type 1 IFN production.

We next examined whether YWHAZ impaired IRF3 dimerization following activation of RLR signaling. As expected, YWHAZ overexpression reduced the interaction between HA-IRF3 and Flag-IRF3 in a dose-dependent manner in HEK-293T cells (Fig. 4D). Accordingly, a reverse Co-IP experiment with anti-Flag was also performed and showed the same results (Fig. 4E). Taken together, these findings demonstrated that YWHAZ protein suppressed TBK1-IRF3 complex formation and IRF3 dimerization through binding IRF3, thereby inhibiting IRF3-mediated signaling.

### YWHAZ inhibited the nuclear translocation of IRF3

**Fig 5:**
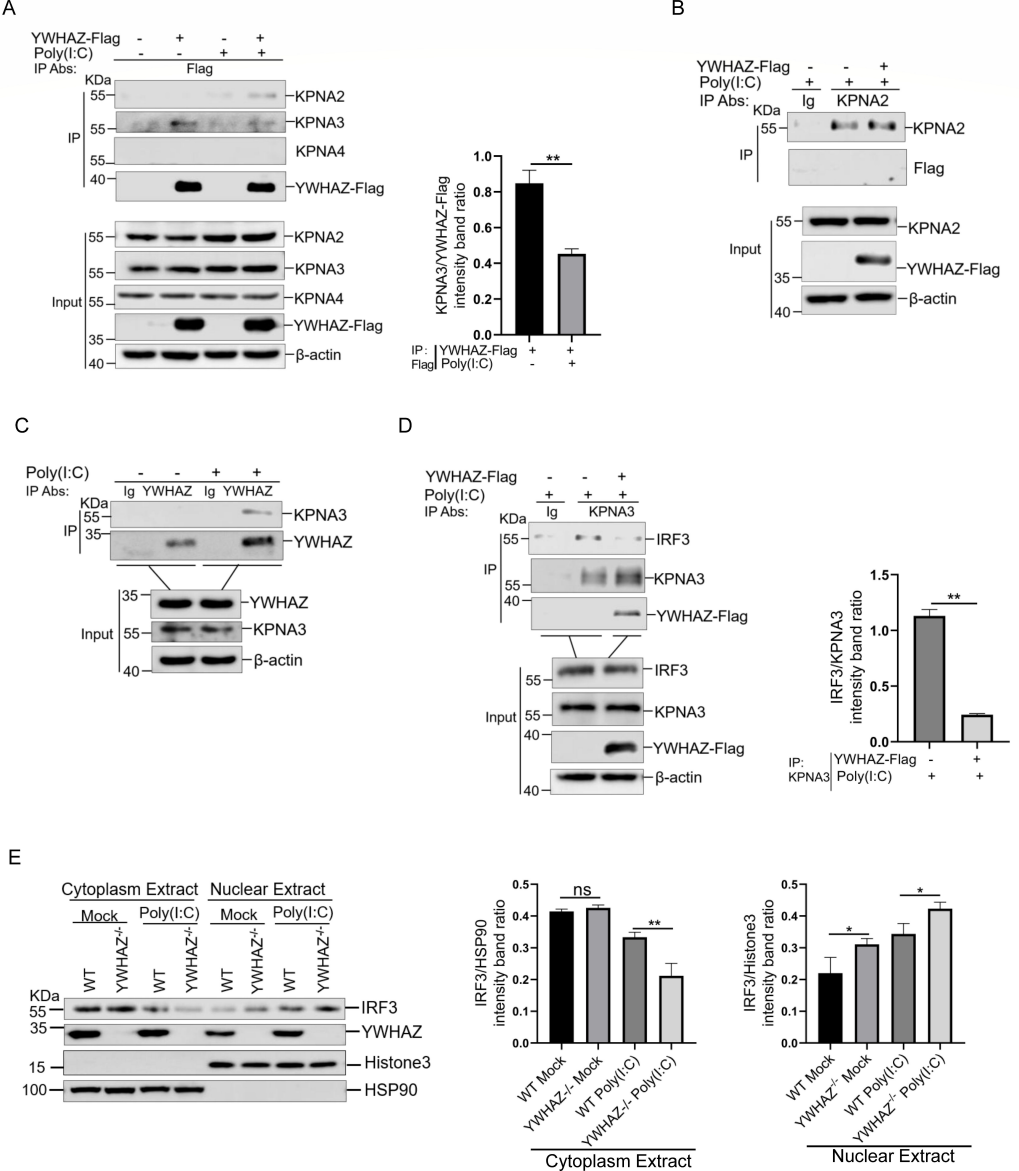
YWHAZ inhibited IRF3 nuclear translocation. (**A**) HEK-293T cells were transfected with the EV (*pCMV-3Tag-3a*) or *pCMV-YWHAZ-Flag* plasmid*s*. At 24 hpt, cells were transfected with poly(I:C) for 12 h. The cells transfected with an empty vector were used as a negative control. Cells were harvested and used for the Co-IP with anti-Flag antibody. WCL and immunoprecipitates were analyzed by western blotting with the indicated antibodies. Representative data from three independent experiments. Band intensity of the KPNA3 protein was quantified using ImageJ software and normalized to that of YWHAZ-Flag in the immunoprecipitation complexes. Data were shown as mean ± SD (*n* = 3). Student’s *t* test was performed. **P < 0.01. (**B**) HEK-293T cells were transfected with the EV (*pCMV-3Tag-3a*) or *pCMV-YWHAZ-Flag* plasmid*s*. At 24 hpt, cells were transfected with poly(I:C) for 12 h. The cells transfected with an empty vector were used as a negative control. Cells were harvested and used for the Co-IP with control IgG or anti-KPNA2 antibodies. WCL and immunoprecipitates were analyzed by western blotting with the indicated antibodies. Representative data from three independent experiments. (**C**) HEK-293T cells were transfected with poly(I:C) for 12 h. Cells were harvested and used for the Co-IP with control IgG or anti-YWHAZ antibodies. WCL and immunoprecipitates were analyzed by western blotting with the indicated antibodies. Representative data from three independent experiments. (**D**) A549 cells were transfected with the EV (*pCMV-3Tag-3a*) or *pCMV-YWHAZ-Flag* plasmid*s*. At 24 hpt, cells were transfected with poly(I:C) for 12 h. The cells transfected with an empty vector were used as a negative control. Cells were harvested and used for the Co-IP with control IgG or anti-KPNA3 antibodies. WCL and immunoprecipitates were analyzed by western blotting with the indicated antibodies. Representative data from three independent experiments. Band intensity of IRF3 protein was quantified using ImageJ software and normalized to that of KPNA3 in the immunoprecipitation complexes. Data were shown as mean ± SD (*n* = 3). Student’s *t* test was performed. ** *P* < 0.01. The cells transfected with an empty vector were used as a negative control. (**E**) WT and YWHAZ^−/−^ HEK-293T cells were transfected with poly(I:C) for 12 h, and then the cells were collected and subjected to nuclear fractionation. The IRF3, phosphorylated IRF3, YWHAZ, Histone3, HSP90, and β-actin were analyzed by western blotting. Histone 3 and HSP90 were analyzed as fraction loading controls. Representative data from three independent experiments. The band intensities of IRF3 in the cytoplasmic and nuclear fractions were quantified by densitometric analysis using ImageJ software, normalized to HSP90 and Histone H3, respectively. Data were shown as mean ± SD (*n* = 3). Student’s *t* test was performed. ns, not significant. * *P* < 0.05; ** *P* < 0.01.

Nuclear translocation of IRF3 is tightly regulated by the NLS and nuclear export sequence (14). KPNA proteins, including KPNA2, KPNA3, and KPNA4, were reported to facilitate nuclear localization of IRF3 through interacting with IRF3 (18, 19). To clarify the mechanism by which YWHAZ impacts type 1 IFNs production via targeting IRF3, we determined whether YWHAZ impaired the activity of IRF3 via KPNA family proteins. The interactions of YWHAZ with KPNA2, KPNA3, and KPNA4 were examined by Co-IP experiments. Results showed that KPNA2 and KPNA3 interacted with YWHAZ, but not KPNA4 (Fig. 5A). Reverse Co-IP assays using anti-KPNA2 antibody were also performed, and YWHAZ protein was not coimmunoprecipitated by KPNA2 in poly(I:C)-stimulated HEK-293T cells (Fig. 5B). Therefore, YWHAZ might not effectively interact with KPNA2 in HEK-293T cells. But the endogenous interaction between YWHAZ and KPNA3 was confirmed via Co-IP experiments using anti-YWHAZ antibody (Fig. 5C). Importantly, the association between KPNA3 and IRF3 was disrupted in A549 cells expressing YWHAZ-Flag upon poly(I:C) treatment (Fig. 5D). This finding suggested that YWHAZ might inhibit IRF3 nuclear translocation by interfering with the KPNA3-IRF3 interaction. Moreover, our results confirmed that YWHAZ inhibited the phosphorylation and dimerization of IRF3. We further detected whether YWHAZ disrupted the nuclear translocation of IRF3 induced by poly(I:C) in YWHAZ^−/−^ cells. Nuclear-cytoplasmic separation analysis indicated that knockout of YWHAZ promoted poly(I:C) triggered-IRF3 nuclear translocation (Fig. 5E). This was consistent with our previous confocal analysis showing that YWHAZ expression significantly inhibited IRF3 nuclear translocation (Fig. 3G). Collectively, these data show that YWHAZ inhibited IRF3 nuclear translocation through directly inhibiting IRF3 phosphorylation-dependent dimerization and disrupting the KPNA3-IRF3 interaction.

### Knockdown of IRF3 eliminated the inhibitory effect of YWHAZ on type 1 IFN response

**Fig 6:**
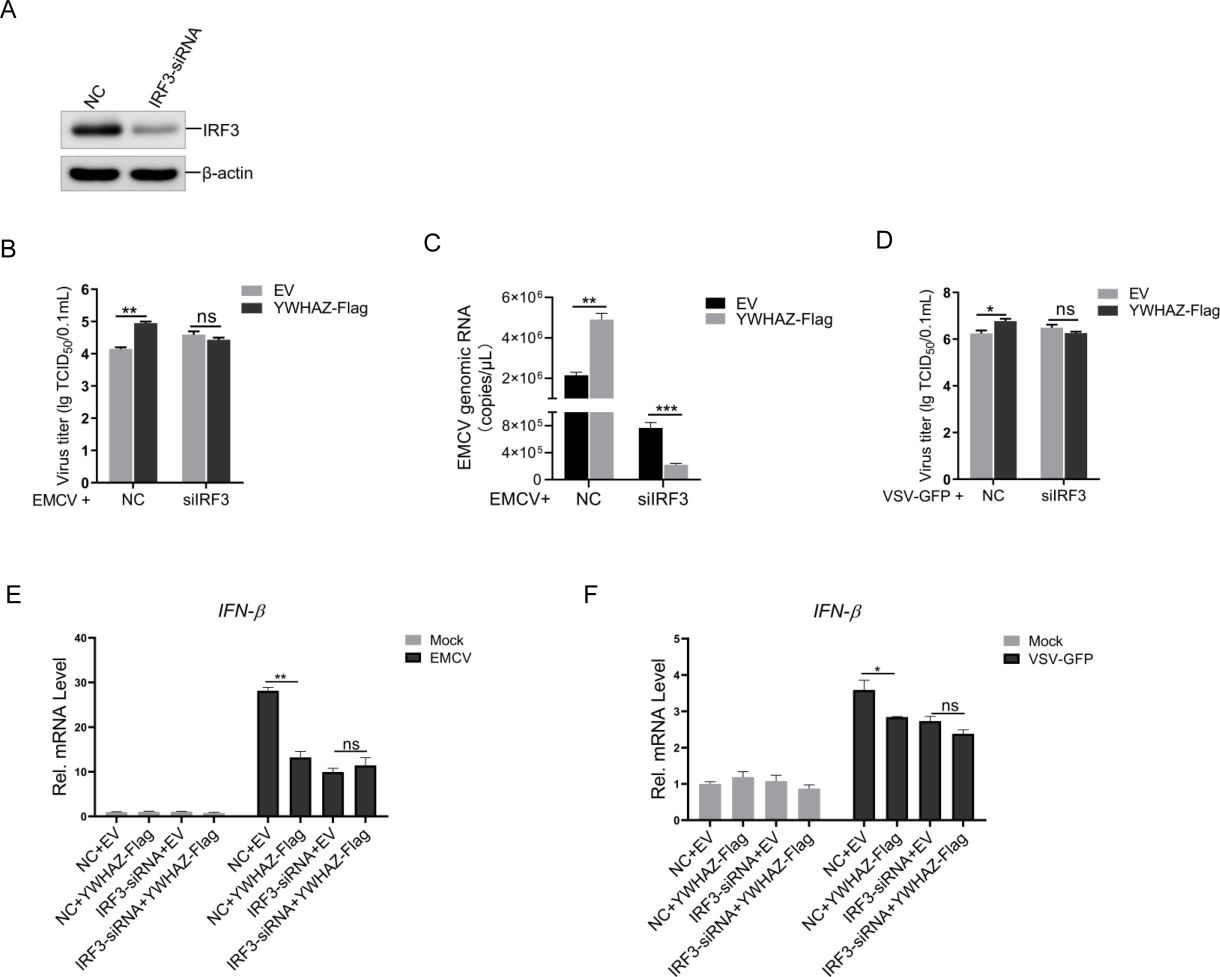
Knockdown of IRF3 eliminated the inhibitory effect of YWHAZ on type 1 IFN response. (**A**) HEK-293T cells were transfected with control siRNA (NC) or siRNA targeting IRF3 (IRF3-siRNA) for 36 h. The protein expression of IRF3 and β-actin was analyzed by western blotting. (**B and C**) HEK-293T cells were transfected with control siRNA (NC) or siRNA targeting IRF3 (IRF3-siRNA). At 12 hpt, cells were transfected with EV (*pCMV-3Tag-3a*) or *pCMV-YWHAZ-Flag* plasmid*s* for another 12 h. Then, cells were infected with EMCV (MOI = 0.01) for 24 h. The TCID_50_ and Q-PCR assays were used to determine the titers (**B**) and viral RNA copy numbers (**C**) of EMCV, respectively. EV, empty vector. Representative data from three independent experiments. Data were shown as mean ± SD (*n* = 3). Student’s *t* test was performed. ns, not significant. **P < 0.01. **P < 0.001. (**D**) HEK-293T cells were transfected with control siRNA (NC) or siRNA targeting IRF3 (IRF3-siRNA). At 12 hpt, cells were transfected with EV (*pCMV-3Tag-3a*) or *pCMV-YWHAZ-Flag* plasmid*s* for another 12 h. Then, cells were infected with VSV-GFP (MOI = 0.001) for 12 h. The TCID_50_ assay was used to determine the titers of VSV-GFP. EV, empty vector. Representative data from three independent experiments. Data were shown as mean ± SD (*n* = 3). Student’s *t* test was performed. ns, not significant. *P < 0.05. (**E and F**) HEK-293T cells were transfected with control siRNA (NC) or siRNA targeting IRF3 (IRF3-siRNA). At 12 hpt, cells were transfected with EV (*pCMV-3Tag-3a*) or *pCMV-YWHAZ-Flag* plasmids for another 12 h. The cells were then infected with EMCV (MOI = 0.01) for 24 h or VSV-GFP (MOI = 0.001) for 12 h. The cells were used to analyze the mRNA level of IFN-β by Q-PCR. EV, empty vector. Representative data from three independent experiments. Data were shown as mean ± SD (*n* = 3). Student’s *t* test was performed. ns, not significant. *P < 0.05; **P < 0.01.

To confirm that YWHAZ is dependent on IRF3 rather than other viral sensor pathways to suppress type 1 IFN response, we designed and synthesized siRNA targeting human IRF3 and transfected them into HEK-293T cells. Western blotting analysis showed that the expression of endogenous IRF3 protein was markedly decreased in cells transfected with IRF3 siRNA compared with the control groups (Fig. 6A). Q-PCR and virus titer analysis were used to monitor the effect of YWHAZ on EMCV proliferation in IRF3-knockdown cells. We observed that knockdown of IRF3 abrogated the enhanced EMCV replication by YWHAZ (Fig. 6B and C). Likewise, knockdown of IRF3 in HEK-293T cells led to the abrogation of the enhanced VSV-GFP replication by YWHAZ (Fig. 6D). The Q-PCR analysis showed that YWHAZ-Flag significantly decreased EMCV-induced IFN-β mRNA levels in HEK-293T cells transfected with siRNA (NC), and the inhibitory effect of YWHAZ on IFN-β expression was reversed in cells with IRF3 expression knocked down (Fig. 6E). Similarly, in VSV-GFP-infected HEK-293T cells, knockdown of IRF3 weakened the inhibitory effect of YWHAZ on VSV-induced IFN-β expression (Fig. 6F). Taken together, our data show that YWHAZ mainly targeted IRF3 to inhibit the IFNs response induced by RNA viruses.

### The 124–184 aa of YWHAZ was critical for the suppression of type 1 IFNs

**Fig 7:**
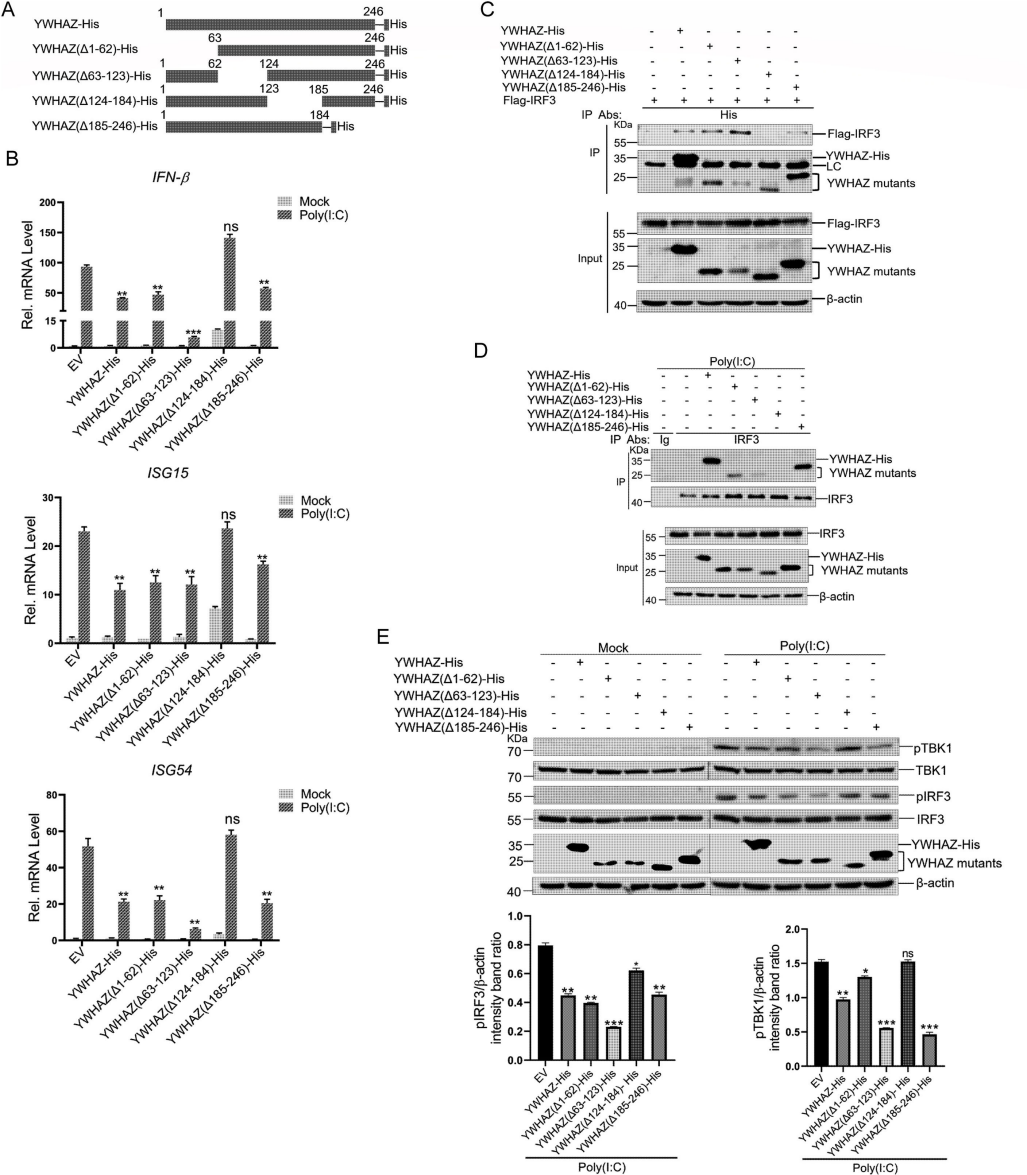
The 124–184 aa of YWHAZ was essential for suppression of type 1 IFNs. (**A**) Schematic representation of full YWHAZ (YWHAZ-His) and truncated mutants of YWHAZ (YWHAZ(Δ1-62)-His, YWHAZ(Δ63-123)-His, YWHAZ(Δ124-184)-His, and YWHAZ(Δ185-246)-His). (**B**) HEK-293T cells were transfected with EV (*pcDNA3.1-His*) or YWHAZ-His or YWHAZ mutants expression plasmids. At 24 hpt, cells were transfected with poly(I:C) or mock-transfected for 24 h. The cells transfected with an empty vector were used as a negative control. The mRNA expression of IFN-β, ISG15, and ISG54 was analyzed by Q-PCR. EV, empty vector. Representative data from three independent experiments. Data were shown as mean ± SD (*n* = 3). One-way ANOVA with Tukey’s multiple-comparison test was performed. ns, not significant; **P < 0.01; ***P < 0.001. (**C**) HEK-293T cells were cotransfected with EV (*pcDNA3.1-His*) or YWHAZ-His or YWHAZ truncated mutants expressing plasmids and Flag-IRF3 plasmids for 36 h. Cells were harvested and used for the Co-IP with anti-His antibody. Whole-cell lysates and immunoprecipitates were analyzed by western blotting with the indicated antibodies. LC denotes the anti-His antibody light chain (25 kDa). Representative data from three independent experiments. (**D**) HEK-293T cells were transfected with EV (*pcDNA3.1-His*) or YWHAZ-His or YWHAZ truncated mutants expressing plasmids for 24 h, followed by transfection of poly(I:C) for 12 h. The cells transfected with an empty vector were used as a negative control. Cells were harvested and used for the Co-IP with control IgG or anti-IRF3 antibodies. Whole-cell lysates and immunoprecipitates were analyzed by western blotting with the indicated antibodies. Representative data from three independent experiments. (**E**) HEK-293T cells were transfected with EV (*pcDNA3.1-His*) or YWHAZ-His or YWHAZ truncated mutants expressing plasmids for 24 h followed by transfection of poly(I:C) for 12 h. The cells transfected with an empty vector were used as a negative control. The TBK1, phosphorylated TBK1, IRF3, phosphorylated IRF3, YWHAZ-His, YWHAZ mutants, and β-actin were analyzed by western blotting. Band intensity of phosphorylated TBK1 and IRF3 protein was determined by densitometric scanning using ImageJ after normalization to β-actin expression. Representative data from three independent experiments. Data were shown as mean ± SD (*n* = 3). One-way ANOVA with Tukey’s multiple-comparison test was performed. ns, not significant; *P < 0.05; **P < 0.01; ***P < 0.001.

To characterize the critical region of YWHAZ inhibiting type 1 IFN production, we designed and constructed the plasmids expressing full-length YWHAZ and truncated mutants of YWHAZ, including YWHAZ-His, YWHAZ(Δ1–62)-His, YWHAZ(Δ63–123)-His, YWHAZ(Δ124–184)-His, and YWHAZ(Δ185–246)-His (Fig. 7A). As is shown in Fig. 7B, deletion of YWHAZ aa 124–184 abrogated YWHAZ-mediated suppressive effect on poly(I:C)-induced IFN-β, ISG15, and ISG54 expression in HEK-293T cells (Fig. 7B). Deletion of the aa 1–62, 63–123, or 185–246 region of YWHAZ did not abrogate the YWHAZ-mediated suppressive effect on IFN-β and ISGs expression (Fig. 7B). The interaction between YWHAZ truncated mutants and IRF3 was examined as well. Co-IP assays showed that deletion of aa 124–184 in YWHAZ abolished the interaction between YWHAZ with IRF3 (Fig. 7C). Consistently, Co-IP experiments using anti-IRF3 antibody indicated that YWHAZ(Δ124–184)-His did not interact with IRF3 in HEK-293T cells (Fig. 7D). Western blotting analysis showed that deletion of aa 124–184 in YWHAZ failed to suppress poly(I:C)-induced TBK1 and IRF3 phosphorylation (Fig. 7E). These results confirm that the 124–184 aa of YWHAZ was essential for suppression of type 1 IFNs by targeting IRF3.

### YWHAZ suppressed type 1 IFN production independently of its self-dimerization

**Fig 8:**
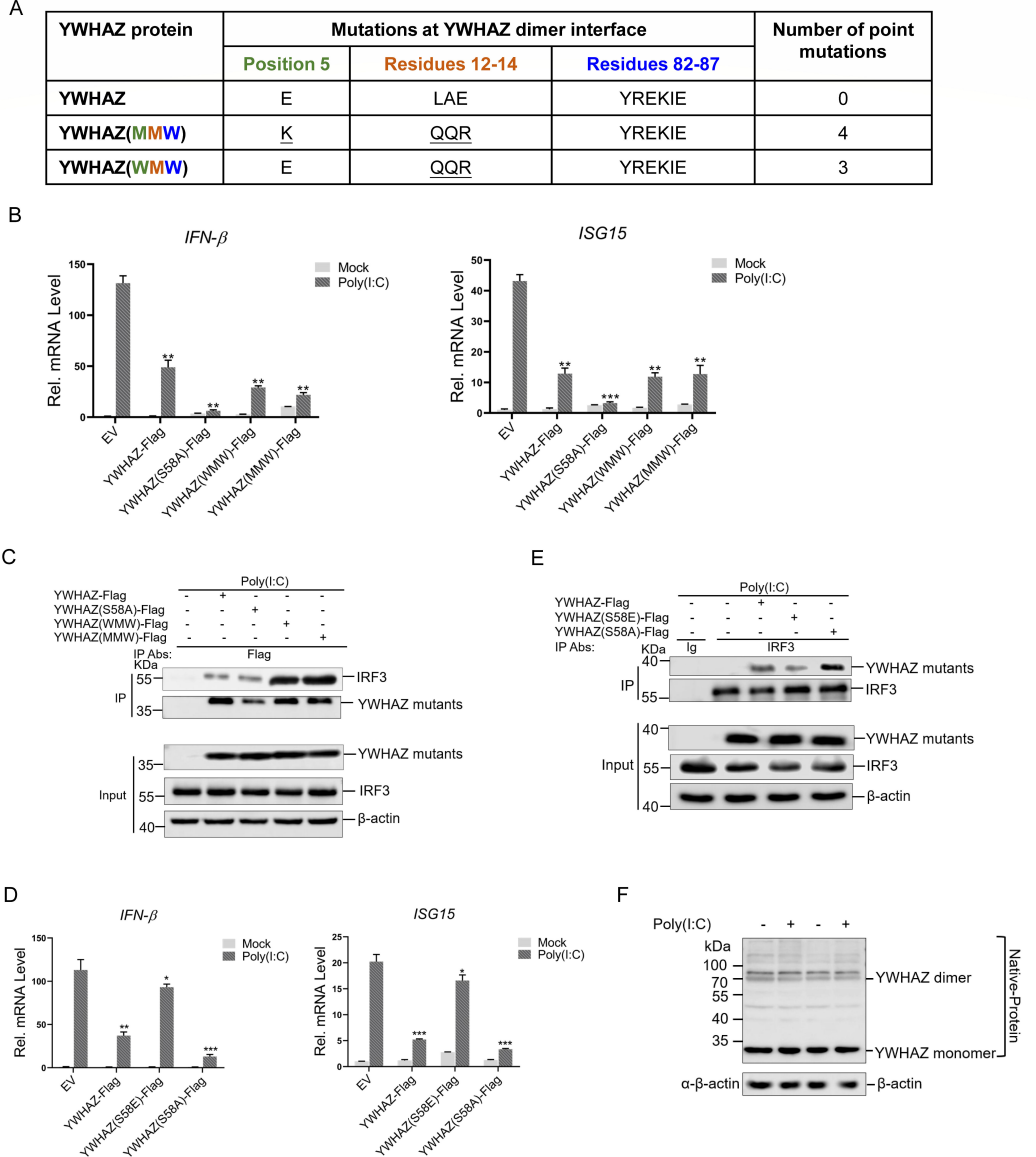
YWHAZ-mediated type 1 IFN suppression was independent of its self-dimerization. (**A**) Designation of dimer-deficient YWHAZ mutants (mutated residues underlined). (**B**) HEK-293T cells were transfected with EV (*pCMV-3Tag-3a*) or YWHAZ-Flag or dimer-deficient YWHAZ mutants expression plasmids. At 24 hpt, cells were transfected with poly(I:C) or mock-transfected for 12 h. The cells transfected with an empty vector were used as a negative control. The mRNA expression of IFN-β and ISG15 was analyzed by Q-PCR. EV, empty vector. Representative data from three independent experiments. Data were shown as mean ± SD (*n* = 3). One-way ANOVA with Tukey’s multiple-comparison test was performed. **P < 0.01; ***P < 0.001. (**C**) HEK-293T cells were transfected with EV (*pCMV-3Tag-3a*) or YWHAZ-Flag or dimer-deficient YWHAZ mutants expressing plasmids for 24 h followed by transfection of poly(I:C) for 12 h. The cells transfected with an empty vector were used as a negative control. Cells were harvested and used for the Co-IP with anti-Flag antibody. WCL and immunoprecipitates were analyzed by western blotting with the indicated antibodies. Representative data from three independent experiments. (**D**) HEK-293T cells were transfected with EV (*pCMV-3Tag-3a*), YWHAZ-Flag, YWHAZ(S58E)-Flag, or YWHAZ(S58A)-Flag mutants expressing plasmids. At 24 hpt, cells were transfected with poly(I:C) for an additional 12 h. The cells transfected with an empty vector were used as a negative control. The mRNA expression of IFN-β and ISG15 was analyzed by Q-PCR. EV, empty vector. Representative data from three independent experiments. Data were shown as mean ± SD (*n* = 3). One-way ANOVA with Tukey’s multiple-comparison test was performed. *P < 0.05; **P < 0.01; ***P < 0.001. (**E**) HEK-293T cells were transfected with EV (*pCMV-3Tag-3a*), YWHAZ-Flag, YWHAZ(S58E)-Flag, or YWHAZ(S58A)-Flag mutants expressing plasmids. At 24 hpt, cells were transfected with poly(I:C) for an additional 12 h. The cells transfected with an empty vector were used as a negative control. Cells were harvested and used for the Co-IP with control IgG or anti-IRF3 antibody. WCL and immunoprecipitates were analyzed by western blotting with the indicated antibodies. Representative data from three independent experiments. (**F**) HEK-293T cells were transfected with poly(I:C) for 12 h. Native cell lysates were used to analyze the YWHAZ monomer and dimer, and denatured protein productions were used to analyze YWHAZ-Flag and β-actin by western blotting with the indicated antibodies. Representative data from three independent experiments.

YWHAZ functions primarily as a dimer, serving as a scaffold protein to regulate key signaling pathways (3, 32). Dimer-deficient mutants of YWHAZ, containing seven mutations predominantly located at the dimer interface, have been previously constructed and studied (33). Each mutated motif (residue 5, residues 12–14, and residues 82–87) was denoted by W (wild type) or M (mutated) (Fig. 8A) (34). The study indicated that both WMW and MMW mutants achieved monomerization over a very wide range of concentrations (33). Moreover, phosphorylation of YWHAZ Ser58 is critical for the stability of YWHAZ dimers (35, 36). Therefore, we constructed plasmids expressing YWHAZ mutants, including YWHAZ(WMW)-Flag, YWHAZ(MMW)-Flag, and YWHAZ(S58A)-Flag, and investigated whether dimer deficiency in YWHAZ affects its immunosuppressive function. As is shown in Fig. 8B, S58A, WMW, and MMW mutants in YWHAZ and wild-type YWHAZ all suppressed IFN-β and ISG15 expression. Meanwhile, YWHAZ-Flag, YWHAZ(WMW)-Flag, YWHAZ(MMW)-Flag, and YWHAZ(S58A)-Flag interacted with IRF3 (Fig. 8C). Notably, these mutants demonstrated a stronger inhibitory effect on the expression of IFN-β and ISG15 compared to the wild type. Specifically, the phosphorylation-deficient mutant YWHAZ(S58A) exhibited a more pronounced inhibition of IFN-β expression. To examine whether phosphorylation at YWHAZ S58 regulates IRF3 signaling, we generated a YWHAZ(S58E) plasmid to simulate constitutive S58 phosphorylation and evaluated the impact of S58 phosphorylation on IFN-β and ISG15 expression by Q-PCR. The YWHAZ(S58A) mutant reduced IFN-β and ISG15 expression more potently than wild-type YWHAZ, whereas the S58E mutant showed a markedly diminished ability to inhibit IFN-β and ISG15 expression (Fig. 8D). Consistently, the interaction between YWHAZ(S58E) and IRF3 was significantly weaker than that of wild-type YWHAZ or the S58A mutant (Fig. 8E). These results indicated that phosphorylation at S58 impaired YWHAZ’s capacity to suppress IRF3 signaling. Furthermore, western blotting analysis revealed that dimerization of YWHAZ had not been significantly changed by poly(I:C) treatment (Fig. 8F). These data together suggested that the ability of YWHAZ to inhibit IRF3 signaling is independent of its phosphorylation at S58 and dimerization.

Taken together, we identified YWHAZ protein as a negative regulator of type 1 IFN production upon infection with RNA viruses. YWHAZ interacted with IRF3 to inhibit the formation of the TBK1-IRF3 complex, the phosphorylation and dimerization of IRF3, as well as the subsequent nuclear translocation. YWHAZ also impaired the interaction between KPNA3 and IRF3 to inhibit the nuclear translocation of IRF3. Residues 124–184 of YWHAZ were critical for YWHAZ-mediated suppression of type 1 IFNs, and this inhibitory effect is independent of phosphorylation at S58 and self-dimerization of YWHAZ.

## DISCUSSION

Upon viral infection, the host’s PRRs detect viral nucleic acids. Among these, cytosolic RLRs, such as RIG-I, MDA5, and LGP2, are responsible for sensing viral RNA. dsRNA-binding RIG-I/MDA5 results in the release of CARD (37). RIG-I/MDA5 then recruits the adaptor protein MAVS through its CARD, which subsequently assembles a signaling platform to recruit and activate downstream signaling molecules TBK1 and IRF3 (15, 38). The TBK1-IRF3 signaling cascade is essential for type 1 IFN production. IRF3 is directly phosphorylated by TBK1 and undergoes dimerization and nuclear translocation to initiate transcription of type 1 IFNs (14, 39). As a key factor in type 1 IFNs signaling, activation of IRF3 must be tightly regulated to prevent dysregulated or excessive immune responses. Nevertheless, the mechanisms regulating IRF3 activation both in the resting state and during viral infection are not yet fully understood. In this study, we found that overexpression of YWHAZ protein inhibited IFN-β production induced by RNA viruses (such as EMCV and VSV) (Fig. 1A and B). In contrast, knockout of the YWHAZ gene significantly inhibited the EMCV and VSV replication and increased IFN-β production induced by poly(I:C) (Fig. 2A through E). Mechanistically, YWHAZ impaired TBK1-IRF3 complex formation, inhibited IRF3 phosphorylation and dimerization, and disrupted KPNA3-IRF3 interaction, thereby blocking IRF3 nuclear translocation. Interestingly, we found that the expression of YWHAZ in mock-infected cells could also significantly reduce the production of IFN-β induced by EMCV (Fig. 1A). Inversely, in the YWHAZ^−/−^ cells, the phosphorylation of IRF3 and the production of poly(I:C)-induced IFN-β and ISGs were significantly upregulated (Fig. 2E and G). Furthermore, in the absence of RLR agonists, YWHAZ also interacted with IRF3 (Fig. 3A, C and D). These results indicated that in the resting state, YWHAZ constitutively interacts with IRF3 and acts as a safeguard to prevent its aberrant activation. However, upon viral infection, YWHAZ switches its role to function as a brake, moderating IRF3 activation to maintain an appropriate immune response level. This study revealed a new mechanism by which the YWHA family protein regulates antiviral immune responses.

Emerging evidence has revealed that YWHAZ plays various roles in numerous signal transduction pathways and is involved in the occurrence and progression of various tumors as an oncogene (5, 40). For example, YWHAZ modulated the PI3K/AKT signaling pathway and thereby regulated the expression of cell cycle and apoptosis-related proteins by inducing phosphorylation of AKT (41). YWHAZ mediated miR-22-induced activation of the caspase signaling pathway, thereby triggering tumor cell apoptosis in hepatocellular carcinoma (41). One study provided evidence that YWHAZ might mediate tumor immune response via Stat3 signaling (9). Moreover, YWHAZ is involved in TLR signaling. Interestingly, YWHAZ exhibited divergent modulation of TLR2 and TLR4 signaling pathways: it suppressed TLR2-mediated NF-κB activation, thereby attenuating pro-inflammatory cytokine production while promoting TLR4-dependent NF-κB signaling (11). The isoforms of YWHA protein, YWHAE and YWHAS, impaired TLR-mediated production of proinflammatory cytokine (42). Therefore, the same YWHA and among various YWHA isoforms existed differences in functionality in the process of mediating different signaling pathways. Our data further indicated that the multiple mechanisms of the molecular chaperone YWHA proteins in regulating antiviral innate immunity. The combined effects of different YWHA family members on the RLR signaling pathway may ensure that immune signaling is properly controlled and does not overrespond.

YWHAZ forms homodimers or heterodimers that interact with various proteins through their amino-terminal α-helical regions (43). The function of YWHAZ can be regulated by interference of its dimerization (44). Monomerization of YWHAZ protein can also be induced by point mutations of residues at the dimeric interface, as in the case of YWHAZ S58, WMW, and MMW mutants resembling the phosphorylated YWHAZ monomer (33, 45). We observed that YWHAZ suppressed type 1 IFN production independently of its self-dimerization (Fig. 8). The poly(I:C) treatment did not induce an obvious change in the dimerization of YWHAZ in HEK-293T cells (Fig. 8D). Similar to the wild-type YWHAZ, dimer-deficient mutants of YWHAZ also interacted with IRF3 (Fig. 8C). Therefore, RLR signal transduction may not involve the dimer monomer equilibrium of YWHAZ. On the other hand, YWHAZ interacts predominantly with other target proteins through a phosphorylated serine/threonine motif. Structural studies of YWHA-nonphosphorylated peptide complex indicated that YWHA proteins can also interact with nonphosphorylated hydrophobic peptide (5, 46). Our results demonstrated that YWHAZ interacted with IRF3 both in the presence and absence of poly(I:C), revealing the association of YWHAZ with nonphosphorylated IRF3 (Fig. 3). The versatility of YWHAZ binding target protein is consistent with the prevalent regulatory effects of YWHAZ mediating different cellular processes. Thus, we identified a new host factor that regulates the function of IRF3 during viral infection of RNA viruses. YWHAZ suppressed the production of type 1 interferon induced by IRF3 through binding IRF3 using 124–184 aa of YWHAZ (Fig. 7). Notably, our data demonstrated that YWHAZ(Δ63-123)-His mutant exhibited a stronger binding affinity for IRF3 and a more potent ability to suppress IFN-β expression (Fig. 7B and C). Structural analysis using the InterPro and RCSB PDB databases indicated that the YWHAZ 63–123aa segment includes several amino acids that are crucial for the dimerization interface. Because YWHAZ inhibited IRF3 signaling independently of dimerization (Fig. 8), deletion of the 63–123 aa might enhance its ability to antagonize IRF3 signaling by disrupting dimer formation. Obviously, YWHAZ may regulate the IRF3 signaling pathway through different mechanisms in the absence of viral infection.

**Fig 9:**
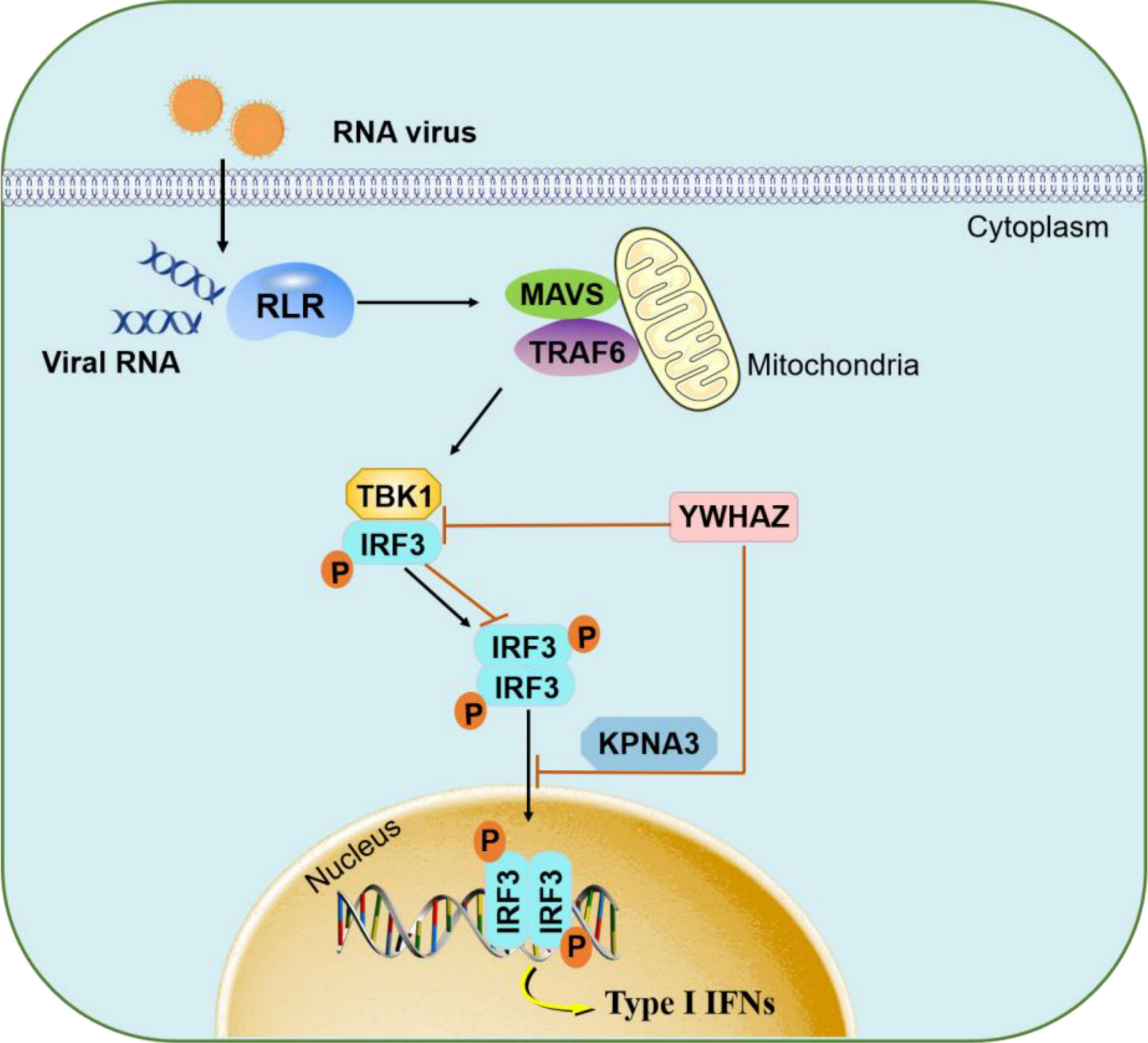
Schematic model of YWHAZ inhibits innate immune responses to viral infections by inhibiting IRF3 signaling pathway. YWHAZ protein was a negative regulator of type 1 IFN production upon infection with RNA viruses. YWHAZ interacted with IRF3 to inhibit the formation of the TBK1-IRF3 complex, the phosphorylation and dimerization of IRF3, as well as the subsequent nuclear translocation. YWHAZ also impaired the interaction between KPNA3 and IRF3 to inhibit the nuclear translocation of IRF3.

In conclusion, we first proposed that YWHAZ was a negative regulator of RNA virus infection-induced IFN-β production in the RLRs signaling pathway. YWHAZ interrupted TBK1-IRF3 complex formation and phosphorylation and the dimerization of IRF3. YWHAZ also inhibited IRF3 nuclear translocation by disrupting the KPNA3-IRF3 interaction (Fig. 9). Our data uncovered the mechanism used by YWHAZ to negatively regulate host antiviral innate immunity. These findings enhanced our understanding of how YWHA proteins mediate innate immune responses.

## Data Availability

All data are available within the article.

## References

[1] Morrison DK. 2009. The 14-3-3 proteins: integrators of diverse signaling cues that impact cell fate and cancer development. Trends Cell Biol 19:16–23. doi:10.1016/j.tcb.2008.10.003 19027299 PMC3073487

[2] Liu J, Cao S, Ding G, Wang B, Li Y, Zhao Y, Shao Q, Feng J, Liu S, Qin L, Xiao Y. 2021. The role of 14-3-3 proteins in cell signalling pathways and virus infection. J Cell Mol Med 25:4173–4182. doi:10.1111/jcmm.16490 33793048 PMC8093981

[3] Obsilova V, Obsil T. 2022. Structural insights into the functional roles of 14-3-3 proteins. Front Mol Biosci 9:1016071. doi:10.3389/fmolb.2022.1016071 36188227 PMC9523730

[4] Liu HM, Loo Y-M, Horner SM, Zornetzer GA, Katze MG, Gale M Jr. 2012. The mitochondrial targeting chaperone 14-3-3ε regulates a RIG-I translocon that mediates membrane association and innate antiviral immunity. Cell Host Microbe 11:528–537. doi:10.1016/j.chom.2012.04.006 22607805 PMC3358705

[5] Xu Y, Ren J, He X, Chen H, Wei T, Feng W. 2019. YWHA/14-3-3 proteins recognize phosphorylated TFEB by a noncanonical mode for controlling TFEB cytoplasmic localization. Autophagy 15:1017–1030. doi:10.1080/15548627.2019.1569928 30653408 PMC6526839

[6] Shi L, Wang X, Guo S, Gou H, Shang H, Jiang X, Wei C, Wang J, Li C, Wang L, Zhao Z, Yu W, Yu J. 2024. TMEM65 promotes gastric tumorigenesis by targeting YWHAZ to activate PI3K-Akt-mTOR pathway and is a therapeutic target. Oncogene 43:931–943. doi:10.1038/s41388-024-02959-9 38341472 PMC10959749

[7] Gan Y, Ye F, He XX. 2020. The role of YWHAZ in cancer: a maze of opportunities and challenges. J Cancer 11:2252–2264. doi:10.7150/jca.41316 32127952 PMC7052942

[8] Li J, Xu X, Wang Q, Wang S, Xiong N. 2019. Retracted: 14-3-3ζ promotes gliomas cells invasion by regulating Snail through the PI3K/AKT signaling. Cancer Med 8:783–794. doi:10.1002/cam4.1950 30656845 PMC6382716

[9] Han X, Han Y, Jiao H, Jie Y. 2015. 14-3-3ζ regulates immune response through Stat3 signaling in oral squamous cell carcinoma. Mol Cells 38:112–121. doi:10.14348/molcells.2015.2101 25556369 PMC4332029

[10] Yoneyama M, Kato H, Fujita T. 2024. Physiological functions of RIG-I-like receptors. Immunity 57:731–751. doi:10.1016/j.immuni.2024.03.003 38599168

[11] Schuster TB, Costina V, Findeisen P, Neumaier M, Ahmad-Nejad P. 2011. Identification and functional characterization of 14-3-3 in TLR2 signaling. J Proteome Res 10:4661–4670. doi:10.1021/pr200461p 21827211

[12] Funami K, Matsumoto M, Obuse C, Seya T. 2016. 14-3-3-zeta participates in TLR3-mediated TICAM-1 signal-platform formation. Mol Immunol 73:60–68. doi:10.1016/j.molimm.2016.03.010 27058640

[13] Onomoto K, Onoguchi K, Yoneyama M. 2021. Regulation of RIG-I-like receptor-mediated signaling: interaction between host and viral factors. Cell Mol Immunol 18:539–555. doi:10.1038/s41423-020-00602-7 33462384 PMC7812568

[14] Al Hamrashdi M, Brady G. 2022. Regulation of IRF3 activation in human antiviral signaling pathways. Biochem Pharmacol 200:115026. doi:10.1016/j.bcp.2022.115026 35367198

[15] Liu F, Zhuang W, Song B, Yang Y, Liu J, Zheng Y, Liu B, Zheng J, Zhao W, Gao C. 2023. MAVS-loaded unanchored Lys63-linked polyubiquitin chains activate the RIG-I-MAVS signaling cascade. Cell Mol Immunol 20:1186–1202. doi:10.1038/s41423-023-01065-2 37582970 PMC10542333

[16] Petro TM. 2020. IFN regulatory factor 3 in health and disease. J Immunol 205:1981–1989. doi:10.4049/jimmunol.2000462 33020188

[17] Nigg EA. 1997. Nucleocytoplasmic transport: signals, mechanisms and regulation. Nature 386:779–787. doi:10.1038/386779a0 9126736

[18] Zhang L, Qiu S, Lu M, Huang C, Lv Y. 2020. Nuclear transporter karyopherin subunit alpha 3 levels modulate Porcine circovirus type 2 replication in PK-15 cells. Virology (Auckland) 548:31–38. doi:10.1016/j.virol.2020.06.003 32838944

[19] Dodantenna N, Cha J-W, Chathuranga K, Chathuranga WAG, Weerawardhana A, Ranathunga L, Kim Y, Jheong W, Lee J-S. 2024. The African swine fever virus virulence determinant DP96R suppresses type I IFN production targeting IRF3. Int J Mol Sci 25:2099. doi:10.3390/ijms25042099 38396775 PMC10889005

[20] Cai Z, Zhang M-X, Tang Z, Zhang Q, Ye J, Xiong T-C, Zhang Z-D, Zhong B. 2020. USP22 promotes IRF3 nuclear translocation and antiviral responses by deubiquitinating the importin protein KPNA2. J Exp Med 217:e20191174. doi:10.1084/jem.20191174 32130408 PMC7201923

[21] Jiao P, Ma J, Zhao Y, Jia X, Zhang H, Fan W, Jia X, Bai X, Zhao Y, Lu Y, Zhang H, Guo J, Pang G, Zhang K, Fang M, Li M, Liu W, Smith GL, Sun L. 2024. The nuclear localization signal of monkeypox virus protein P2 orthologue is critical for inhibition of IRF3-mediated innate immunity. Emerg Microbes Infect 13:2372344. doi:10.1080/22221751.2024.2372344 38916407 PMC11229740

[22] Wang R, Zhu Y, Lin X, Ren C, Zhao J, Wang F, Gao X, Xiao R, Zhao L, Chen H, Jin M, Ma W, Zhou H. 2019. Influenza M2 protein regulates MAVS-mediated signaling pathway through interacting with MAVS and increasing ROS production. Autophagy 15:1163–1181. doi:10.1080/15548627.2019.1580089 30741586 PMC6613841

[23] Schmittgen TD, Livak KJ. 2008. Analyzing real-time PCR data by the comparative C_T_ method. Nat Protoc 3:1101–1108. doi:10.1038/nprot.2008.73 18546601

[24] Reed LJ, Muench H. 1938. A simple method of estimating fifty per cent endpoints. Am J Hyg 27:493–497. doi:10.1093/oxfordjournals.aje.a118408

[25] Li S, Zhu Z, Yang F, Cao W, Yang J, Ma C, Zhao Z, Tian H, Liu X, Ma J, Xiao S, Zheng H. 2021. Porcine epidemic diarrhea virus membrane protein interacted with IRF7 to inhibit type I IFN production during viral infection. J Immunol 206:2909–2923. doi:10.4049/jimmunol.2001186 34127522

[26] Kato H, Takeuchi O, Sato S, Yoneyama M, Yamamoto M, Matsui K, Uematsu S, Jung A, Kawai T, Ishii KJ, Yamaguchi O, Otsu K, Tsujimura T, Koh C-S, Reis e Sousa C, Matsuura Y, Fujita T, Akira S. 2006. Differential roles of MDA5 and RIG-I helicases in the recognition of RNA viruses. Nature 441:101–105. doi:10.1038/nature04734 16625202

[27] Kato H, Takeuchi O, Mikamo-Satoh E, Hirai R, Kawai T, Matsushita K, Hiiragi A, Dermody TS, Fujita T, Akira S. 2008. Length-dependent recognition of double-stranded ribonucleic acids by retinoic acid-inducible gene-I and melanoma differentiation-associated gene 5. J Exp Med 205:1601–1610. doi:10.1084/jem.20080091 18591409 PMC2442638

[28] Lian H, Zang R, Wei J, Ye W, Hu M-M, Chen Y-D, Zhang X-N, Guo Y, Lei C-Q, Yang Q, Luo W-W, Li S, Shu H-B. 2018. The zinc-finger protein ZCCHC3 binds RNA and facilitates viral RNA sensing and activation of the RIG-I-like receptors. Immunity 49:438–448. doi:10.1016/j.immuni.2018.08.014 30193849

[29] Hama S, Watanabe-Takahashi M, Nishimura H, Omi J, Tamada M, Saitoh T, Maenaka K, Okuda Y, Ikegami A, Kitagawa A, et al.. 2025. CaMKII-dependent non-canonical RIG-I pathway promotes influenza virus propagation in the acute-phase of infection. mBio 16:e00087-24. doi:10.1128/mbio.00087-24 39601535 PMC11708044

[30] Zhang B, Li M, Chen L, Yang K, Shan Y, Zhu L, Sun S, Li L, Wang C. 2009. The TAK1-JNK cascade is required for IRF3 function in the innate immune response. Cell Res 19:412–428. doi:10.1038/cr.2009.8 19153595

[31] Wang P, Zhao W, Zhao K, Zhang L, Gao C. 2015. TRIM26 negatively regulates interferon-β production and antiviral response through polyubiquitination and degradation of nuclear IRF3. PLoS Pathog 11:e1004726. doi:10.1371/journal.ppat.1004726 25763818 PMC4357427

[32] Ayyasamy R, Fan S, Czernik P, Lecka-Czernik B, Chattopadhyay S, Chakravarti R. 2024. 14-3-3ζ suppresses RANKL signaling by destabilizing TRAF6. J Biol Chem 300:107487. doi:10.1016/j.jbc.2024.107487 38908751 PMC11331427

[33] Sluchanko NN, Sudnitsyna MV, Seit-Nebi AS, Antson AA, Gusev NB. 2011. Properties of the monomeric form of human 14-3-3ζ protein and its interaction with tau and HspB6. Biochemistry 50:9797–9808. doi:10.1021/bi201374s 21978388

[34] Sluchanko NN, Chernik IS, Seit-Nebi AS, Pivovarova AV, Levitsky DI, Gusev NB. 2008. Effect of mutations mimicking phosphorylation on the structure and properties of human 14-3-3ζ. Arch Biochem Biophys 477:305–312. doi:10.1016/j.abb.2008.05.020 18559254

[35] Woodcock JM, Murphy J, Stomski FC, Berndt MC, Lopez AF. 2003. The dimeric versus monomeric status of 14-3-3ζ is controlled by phosphorylation of Ser^58^ at the dimer interface. J Biol Chem 278:36323–36327. doi:10.1074/jbc.M304689200 12865427

[36] Crha R, Kozeleková A, Hofrová A, Iľkovičová L, Gašparik N, Kadeřávek P, Hritz J. 2024. Hiding in plain sight: complex interaction patterns between Tau and 14-3-3ζ protein variants. Int J Biol Macromol 266:130802. doi:10.1016/j.ijbiomac.2024.130802 38492709

[37] Kowalinski E, Lunardi T, McCarthy AA, Louber J, Brunel J, Grigorov B, Gerlier D, Cusack S. 2011. Structural basis for the activation of innate immune pattern-recognition receptor RIG-I by viral RNA. Cell 147:423–435. doi:10.1016/j.cell.2011.09.039 22000019

[38] Hou F, Sun L, Zheng H, Skaug B, Jiang Q-X, Chen ZJ. 2011. MAVS forms functional prion-like aggregates to activate and propagate antiviral innate immune response. Cell 146:448–461. doi:10.1016/j.cell.2011.06.041 21782231 PMC3179916

[39] Taniguchi T, Ogasawara K, Takaoka A, Tanaka N. 2001. IRF family of transcription factors as regulators of host defense. Annu Rev Immunol 19:623–655. doi:10.1146/annurev.immunol.19.1.623 11244049

[40] Huang Y, Li S, Liu Q, Wang Z, Li S, Liu L, Zhao W, Wang K, Zhang R, Wang L, Wang M, William Ali D, Michalak M, Chen X-Z, Zhou C, Tang J. 2022. The LCK-14-3-3ζ-TRPM8 axis regulates TRPM8 function/assembly and promotes pancreatic cancer malignancy. Cell Death Dis 13:524. doi:10.1038/s41419-022-04977-5 35665750 PMC9167300

[41] Chen M, Hu W, Xiong C-L, Qu Z, Yin C-Q, Wang Y-H, Luo C-L, Guan Q, Yuan C-H, Wang F-B. 2016. miR-22 targets YWHAZ to inhibit metastasis of hepatocellular carcinoma and its down-regulation predicts a poor survival. Oncotarget 7:80751–80764. doi:10.18632/oncotarget.13037 27811373 PMC5348352

[42] Butt AQ, Ahmed S, Maratha A, Miggin SM. 2012. 14-3-3ε and 14-3-3σ inhibit Toll-like receptor (TLR)-mediated proinflammatory cytokine induction. J Biol Chem 287:38665–38679. doi:10.1074/jbc.M112.367490 22984265 PMC3493911

[43] Jansen S, Narasimhan S, Cabre Fernandez P, Iľkovičová L, Kozeleková A, Králová K, Hritz J, Žídek L. 2025. Characterization of multiple binding sites on microtubule associated protein 2c recognized by dimeric and monomeric 14-3-3ζ. FEBS J 292:1991–2016. doi:10.1111/febs.17405 39877981 PMC12001206

[44] Gerst F, Kaiser G, Panse M, Sartorius T, Pujol A, Hennige AM, Machicao F, Lammers R, Bosch F, Häring H-U, Ullrich S. 2015. Protein kinase Cδ regulates nuclear export of FOXO1 through phosphorylation of the chaperone 14-3-3ζ. Diabetologia 58:2819–2831. doi:10.1007/s00125-015-3744-z 26363783

[45] Kozeleková A, Náplavová A, Brom T, Gašparik N, Šimek J, Houser J, Hritz J. 2022. Phosphorylated and phosphomimicking variants may differ-a case study of 14-3-3 protein. Front Chem 10:835733. doi:10.3389/fchem.2022.835733 35321476 PMC8935074

[46] Ottmann C, Yasmin L, Weyand M, Veesenmeyer JL, Diaz MH, Palmer RH, Francis MS, Hauser AR, Wittinghofer A, Hallberg B. 2007. Phosphorylation-independent interaction between 14-3-3 and exoenzyme S: from structure to pathogenesis. EMBO J 26:902–913. doi:10.1038/sj.emboj.7601530 17235285 PMC1794388

